# Decoding Dystonia in Autoimmune Disorders: A Scoping Review

**DOI:** 10.5334/tohm.915

**Published:** 2024-12-06

**Authors:** Debayan Dutta, Ravi Yadav

**Affiliations:** 1 Department of Neurology, Shalby Hospital, Jabalpur, Madhya Pradesh 48600, India; 2 Department of Neurology, National Institute of Mental Health and Neurosciences (NIMHANS), Bangalore, Karnataka 560029, India

**Keywords:** dystonia, autoimmune encephalitis, autoimmune dystonia

## Abstract

**Background:**

Dystonia is a common hyperkinetic movement disorder observed in various genetic, infective, drug-induced, and autoimmune disorders. Autoimmune disorders can present with isolated or combined acute or subacute dystonia. The pattern and approach to dystonia in autoimmune disorders are poorly described and have never been established in a structured manner.

**Objective:**

This scoping review aims to summarize all available clinical literature and formulate a pattern and approach to dystonia in different autoimmune disorders.

**Methods:**

We included one hundred and three articles in this scoping review. Most articles identified were case reports or case series.

**Results:**

In this review, we analysed data from 103 articles and summarized the epidemiological, clinical, and diagnostic features of dystonia associated with different autoimmune diseases. We highlight that dystonia can be isolated or combined in various autoimmune conditions and is responsive to immunotherapy. We point out the patterns of dystonia and associated neurological features and investigations that can suggest the underlying autoimmune nature, which can guide the most appropriate treatment.

**Discussion:**

The clinical pattern of dystonia can be a unique feature in many autoimmune disorders. In isolated subacute dystonia, the presence of autoantibodies could have a temporal association, or this is just an epiphenomenon to be evaluated in further research.

**Highlights:**

- Many autoimmune disorders can present with isolated or combined dystonia.
- Subacute onset focal or segmental dystonia (craniocervical dystonia or limb dystonia) or hemidystonia could be secondary to an autoimmune condition and warrants investigations.
- They have a relapsing or progressive course.
- They usually have a good response to early immunotherapy.
- Symptomatic treatment, including botulinum toxin, can be useful in focal dystonia.

## Introduction

Dystonia is a hyperkinetic movement disorder characterized by sustained or intermittent muscle contractions that cause abnormal, often repetitive, movements, postures, or both. The pathogenesis of dystonia involves abnormal dopamine signaling and mitochondrial dysfunction, which lead to dysregulated neurotransmission in the basal ganglia or its circuitry [1]. Dystonia, which was previously classified as a disease of basal ganglia, is now regarded as a ‘network’ disorder that includes other extrapyramidal structures like the cerebellum. There is increased recognition of the cerebellum’s role in the pathogenesis of dystonia, especially since many forms of autoimmune encephalitis are also associated with cerebellar ataxia [2].

The etiology of dystonia is complex and can be divided broadly into hereditary and acquired causes. Among the acquired causes, immune-mediated causes of dystonia are essential because they are reversible and carry an excellent prognosis if treated early. However, the most common etiology of dystonia remains idiopathic even after extensive investigation.

Immune-mediated dystonia is caused by antibodies produced peripherally in the bone marrow which crosses the Blood-Brain barrier or it can be caused by antibodies synthesized intrathecally. The antibodies target different components of neurons, including intranuclear, cytoplasmic components, or synaptic receptors. Clinically, they can be differentiated from hereditary dystonia by their acute to subacute onset, lack of family history, and dramatic response to immunotherapy [3]. Usually, immune-mediated dystonia tends to present as combined dystonia, but when dystonia is the presenting feature or forms the dominant part of the phenomenology, existing literature is scarce regarding the pattern of involvement. In this article, we review the pattern of dystonia in different autoimmune disorders and formulate a clinical approach when dystonia is the sole or dominant presenting feature in a patient with a suspected autoimmune disorder.

## Methods

**Figure 1:**
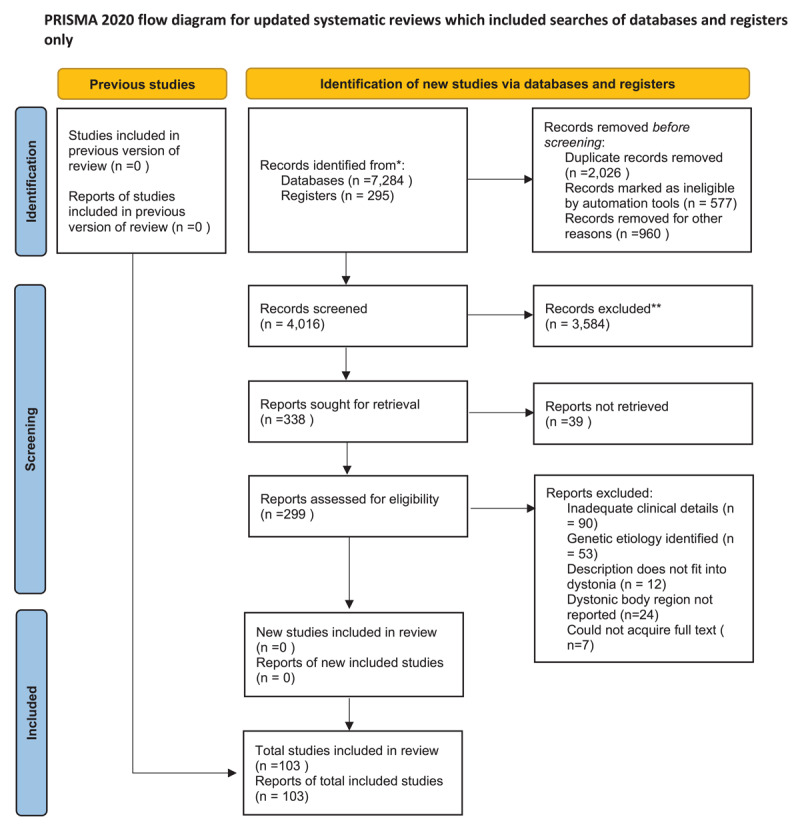
PRISMA flow diagram of the study. *Consider, if feasible to do so, reporting the number of records identified from each database or register searched (rather than the total number across all databases/registers). **If automation tools were used, indicate how many records were excluded by a human and how many were excluded by automation tools. Source: Page MJ, et al. BMJ 2021; 372: n71. doi: 10.1136/bmj.n71. This work is licensed under CC BY 4.0. To view a copy of this license, visit https://creativecommons.org/licenses/by/4.0/.

We searched the literature as of October 2024 using PubMed and MEDLINE to identify relevant articles published between 1965 to 2024. This scoping review was based on the work of Arksey et al [4]. and conducted by the Preferred Reporting Items for Systematic Reviews and Meta-Analyses extension for Scoping Reviews (PRISMA-ScR) guidelines [5](Figure 1).

### Identifying the research question

This scoping review sought to answer the question, “What is the pattern of dystonia in different autoimmune disorders?” The objectives were to review available qualitative and quantitative literature, determine if enough literature has been published for a full systematic review, and provide the building blocks for further research.

### Identifying relevant studies

All the autoimmune conditions where there is existing literature about an association with dystonia and other hyperkinetic movement disorders were included. In October 2024 authors used Pubmed and MEDLINE to search the relevant literature using the following search terms- ***“Movement disorder,” “Dystonia,” “Autoimmune encephalitis (AE),” “NMDA receptor encephalitis,” “LGI Encephalitis,” “Anti Ri Encephalitis,” “Anti IgLON5 associated encephalitis,” “Anti GAD 65 Encephalitis,” “Anti GABA receptor Encephalitis,” “Anti D2R Encephalitis,” “Anti Ma2 Encephalitis,” “Anti PDE10 A Encephalitis,” “Anti CRMP5 Encephalitis”, “Myelin Oligodendrocyte Associated Disease (MOGAD),” “Anti mGluR5 Encephalitis” “Sjogren Syndrome,” “Systemic Lupus Erythematosus (SLE).”*** Only the articles with antibody-positive definite Autoimmune Encephalitis were included in the search strategy; double antibody positive, and seronegative Autoimmune Encephalitis, were excluded. Autoimmune encephalitis was described as per the Graus et al. criteria [6].

### Selection of studies

Two independent reviewers (RY and DD) screened the titles and abstracts for relevant topics to determine whether to consider a full-text review. We identified further articles by cross-referencing. All types of studies, including retrospective observational studies, case series, individual case reports, letters to editors, systematic reviews, and congress posters or oral presentations, were considered for inclusion in this scoping review. Animal studies, non-English articles, and duplicate articles were excluded (Table 1).

**Table 1:** Brief description of different phenotypes of dystonia in different autoimmune disorders.


ANTIBODY	PREVALENCE OF DYSTONIA	AGE OF INVOLVEMENT	PATTERN OF DYSTONIA	OTHER ASSOCIATED NEUROLOGICAL SYMPTOM OR MOVEMENT DISORDER	INVESTIGATION	TREATMENT OUTCOME

**NMDA Receptor Encephalitis**	20%	6–18 years	*Phenotype 1- Classical-* In Children polysymptomatic MD, orofacial lingual dyskinesia, stereotypes. *Phenotype 2*-Monosymptomatic MD Limb or OMD, hemidystonia in adults	Chorea, Steriotypies, Psychiatric and behavioral features, dysautonomia. Oculogyric crisis in children	MRI- Normal or non-specific changes. T2/FLAIR hyperintensity in mesial temporal lobe, cerebral and cerebellar cortex PET- Both hypo and hypermetabolism of the affected region depending on time and severity of presentation. EEG- Generalized Slowing	IVMPS IVIg PLEX Rituximab Good response to IT

**LGI 1 associated encephalitis**	40%	40–70 years	FBDS	Cognitive decline, Insomnia, hyponatremia	MRI – Bitemporal hyperintensity, T2 signal changes in basal ganglia. EEG- normal	IVMPS IVIg Good response to first line IT

**Anti Ri associated Encephalitis**	17- 33%	40–80 years	Craniocervical Dystonia (Jaw closing dystonia, torticollis) Exclusively occurring in the context of breast cancer	Cerebellar ataxia, OMAS	MRI- Normal or T2/FLAIR signal changes in brainstem, upper cervical cord, basal ganglia, mesial temporal lobes.	Good response to symptomatic therapy than IT or tumor removal.

**Anti IgLon5 associated Encephalitis:**	26%	40–90 years	*Phenotype 1* – painful craniocervical dystonia Associated with dystonic tremor and myoclonus of same region. *Phenotype 2 – Subtle* Limb dystonia, upper limb, and finger, never presenting complaint	Chorea, PSP like phenotype, SPS like, facial dyskinesia, cerebellar ataxia	MRI – normal or cerebral atrophy	Craniocervical Dystonia less responsive to IT, required tetrabenazine, Botox Limb dystonia more responsive to IT

**Anti-GAD65 antibody-associated Encephalitis:**	0.03%	5–83 years	*Phenotype 1*- insidious onset lower limb predominant limb dystonia occurring more in 5^th^ decade (47) and older. *Phenotype-2* subacute onset craniocervical dystonia seen in middle aged patients	SPS, Epilepsy, LE, ataxia, cognitive decline, myelopathy Associated with systemic autoimmune disease T1DM, Thyroid disease	MRI – Normal or disproportionate cerebral atrophy EMG –to differentiate from SPS.	Good outcome with IVIg in young Older patient respond to symptomatic therapy more.

**Anti GABA receptor Encephalitis:**	35%	10 month-63 years	Closely follows NMDA encephalitis.	Refractory seizure, cognitive decline	MRI – Multifocal cortical and subcortical FLAIR hyperintensities	Good response to IT, poor response to symptomatic local therapy

**Anti D2R Encephalitis**			Usually hemidystonia with a dystonic tremor Some have oro- mandibular Dystonia. Progress to status Dystonicus	Tics, parkinsonism, psychiatric or sleep disturbance, seizure.	MRI – Bilateral Basal Ganglia FLAIR hyperintensity, striatal necrosis in later stage FDG PET – hypermetabolism of the affected region	Good response to IT (90% had complete or partial resolution), TS and Isolated psychosis group – symptomatic therapy

**Anti PDE 10 A Encephalitis**	Limited Case reports	70 years (range 66–76);	Combined dystonia with other hyperkinetic movement disorders	Encephalopathy Often associated with cancer or follows treatment with immune checkpoint inhibitors (ICI).	MRI- Bilateral T2/FLAIR basal ganglia hyperintensities, leptomeningeal enhancements	Poor response to immunotherapy, cessation of ICI or treatment of cancer.

**Anti Ma2 Encephalitis**	Limited Case reports	26–70 years	Jaw opening dystonia and left upper limb dystonia (as a part of MSA phenotype)	Limbic, diencephalic, brainstem encephalopathy, narcolepsy-cataplexy, eye movement abnormalities (60% had vertical gaze paresis evolved to total external ophthalmoplegia). Usually associated with testicular tumors.	MRI- T2/FLAIR signal changes in the bilateral or unilateral medial temporal lobe, hypothalamus, thalamus, mid-brain, pons, superior and middle cerebellar peduncle. PET – Hypermetabolism in the abnormal area. Low CSF hypocretin level	Poor response to combined immunotherapy and cancer treatment. One-third had a partial response to treatment with the majority of them relapsed later.

**Anti CRMP5**	Limited Case Reports	60–70 years	Limb dystonia associated with other hyperkinetic movements (chorea, ataxia)	Limbic Encephalitis, chorea, ataxia, peripheral neuropathy, myelopathy optic neuritis or retinitis.	MRI – Can be normal or multiple T2/FLAIR high signal foci, mainly involving the basal ganglia, medial temporal lobe, white matter, cerebellum, insula, optic nerve, thalamus, and frontal lobe. PET- Hypometabolism in bilateral caudate, frontoparietal lobes.	Poor response to immunotherapy. However early and aggressive anti-tumor plus immunotherapy is required.

**MOG Antibody Disease**	Limited Case Reports	2–28 years	Combined dystonia including orofacial and limb dystonia associated with encephalopathy, and seizure.	Fever, encephalopathy, seizure, paresis, headache.	MRI- Multifocal T2/FLAIR white matter, cortical, spinal cord hyperintensity	Good response to steroids and other second-line immunotherapy and local symptomatic therapy as required.

**Anti mGluR5**	Limited Case Reports	Second to third decade	Combined generalized and orofacial dystonia with encephalopathy	Limbic Encephalitis, hallucination, cognitive decline, refractory seizure.	MRI – T2/FLAIR hyperintensities in the limbic region or extra limbic regions, meningeal enhancements.	Good response to combined immunotherapy and anti-tumor therapy.

**Sjogren Syndrome**	2.2%	36–57 years	Phenotypes: 1. Late-onset (median 57.5 years) slowly progressive Craniocervical dystonia OR 2. Unilateral painful paroxysmal dystonic attacks of one or both limbs (duration: <2 min, frequency: several times/day)	Cognitive decline, Neuropathy, Aseptic meningitis, cranial neuropathy.	Phenotype 1: MRI: normal, or multiple subcortical T2- hyperintensities Phenotype 2: MRI: ischemic or inflammatory brain/spinal lesions	Phenotype 1: Positive outcome following immunotherapy Phenotype 2: Good outcome with immunotherapy and symptomatic treatments (CBZ/Phenytoin)

**Systemic Lupus Erythematosus (SLE)**		8–65 years	Two phenotypes 1.MR Positive- Middle aged male with complex hyperkinetic movement disorder 2. MR negative – Female, extremes of ages, isolated focal or hemidystonia	MR positive cases- chorea, parkinsonism, myoclonus, athetoid movements.	MRI -contralateral or bilateral Basal ganglia FLAIR changes Antibody panel- ANA (speckled pattern), high positivity of Anti dsDNA, normal C3, C4	MR positive- very good response to IT. MR Negative – Variable response to IT

**Antiphospholipid Antibody Syndrome**		7–76 years	Isolated focal Dystonia or Hemidystonia	Chorea, parkinsonism	MRI – Basal ganglia infarction T2/FLAIR hyperintense lesion Antibody panel – aCL IgG was positive in all patients. B2GP and LA were positive in complex MD cases	Mixed response to IT with anticoagulation

**Neuro Behcet’s Disease**	6%	20–30 years	Paroxysmal painful focal dystonia or hemidystonia	Ataxia, Brainstem signs	MRI – T2/FLAIR high signal in basal ganglia, brain stem, cerebral cortex	Good response to IT and D2 Blocker


A total of 299 articles were identified for further review. Each author independently reviewed each article to determine the quality of the study and information provided. In case of discrepancy, a discussion was held between the two authors to reaffirm the objectives and come to a consensus if the article should be further evaluated. Ultimately, 103 citations were selected for full-text analysis. PRISMA flow chart for the same is included in Figure 1.

### Charting the data

For these 103 articles, we created a standardized extraction form to collect data from the selected articles. It included study design, year of publication, number of patients, antibody/disease association, diagnostic or treatment response, and study findings. Any differences in data extraction were discussed between the reviewers until a consensus was reached.

### Collating, Summarizing, and Reporting Results

We looked at the both qualitative and quantitative results of the article. Each citation was reviewed, and direct quotes and overarching themes were recorded. Overall, 103 papers were selected. **Supplemental Table 1** summarizes all the included studies.

## Results

### A. Dystonia caused by Central Nervous System (CNS) autoimmune diseases

With the discovery of newer antibodies, many mixed hypokinetic and hyperkinetic movement disorders are being increasingly recognized as an autoimmune phenomenon. Both isolated and combined dystonia can be a clinical presentation of these syndromes.

#### 1. NMDA (N-methyl-D-aspartate) receptor Encephalitis

Movement disorder is seen in almost 80% of patients with NMDA (N-methyl-D-aspartate) encephalitis. Patients present with complex polysymptomatic movement disorders, with 20% of patients having dystonia or dyskinesia [7]. Among other movement disorders, chorea, stereotypies, clonic perseverations, abnormal eye movements, parkinsonism and catatonia are common [8,9].

The most common movement disorder is orofacial lingual dyskinesia (OFLD) which is described as complex, involuntary, and repetitive movements of the face and mouth, including grimacing, sucking, and rolling. Duan et al. concluded that OFLD was the second most prevalent movement disorder in children less than 18 years old (second only to choreoathetosis) and the most common movement disorder in patients more than 18 years old [10] [11]. Another study noted that OFLD is the most common movement disorder in all pediatric age groups [12].

The dyskinesia of NMDAR encephalitis has a unique characteristic that differentiates it from other common orofacial dyskinesia like Tardive dyskinesia or oculomasticatory myorrhythmia (OMM). They have a slower frequency (<3–6 Hz), are more widespread, and often persist in unresponsive states [13]. There has been significant inter-rater variability in categorizing these movements, and often, the final category does not do justice to the overall spectrum of hyperkinetic movements. In a video rater study, the kappa value (measurement of interrater reliability) of κ = 0.65 vs 0.39 (controls vs NMDAR encephalitis) was assigned for the dominant phenomenology as dystonia [9] which indicates substantial agreement between the video raters.

Isolated monosymptomatic dystonia at onset is rare in NMDA encephalitis in children. However, it can be the presenting symptom of NMDA encephalitis in adults.

We have noted seven such cases in existing literature where dystonia precedes the onset of the full spectrum of NMDA encephalitis. Distribution of dystonia can be varied like unilateral focal Dystonia (1/7), hemidystonia (1/7), bilateral pseudoathetosis of upper limbs (2/7) [14], or craniocervical Dystonia including blepharospasm (1/7), cervical dystonia with dystonic tremor (1/7) and spasmodic dysphonia (1/7). They all have good responses to immunotherapy [15,16,17].

Tzoulis et al. described a single patient with isolated progressive generalized dystonia with bilateral striatal necrosis with onset at the age of 9 years without any behavioral or cognitive dysfunction. Brain imaging of the patient showed the involvement of the dorsal striatum with corresponding hypometabolism in PET (positron emission tomography). Wilson’s disease, mitochondrial disorders, and DYT syndromes were ruled out using genetic profiling. His CSF NMDA was strongly positive. He received intravenous immunoglobulin (IVIg) without much clinical improvement [18].

Three pediatric patients with craniocervical dystonia, two patients with dystonic spasms, and one patient with oculogyric crisis were described. Oromandibular Dystonia in these children was distinct from the common stereotypical grimacing or pouting [19]. The distribution of dystonic posturing was variable, with the most common being trunk dystonia causing spontaneous opisthotonos posturing [20].

Oculogyric crisis was also noted in many young patients with NMDA encephalitis during acute episodes [14,21].

One article described status dystonicus in one patient with associated choreoathetoid movement [22].

Although not a classical movement disorder, dystonic seizure, including faciobrachial dystonic seizure, had been described in a young female with hyperactivity of ipsilateral insula and basal ganglia on SPECT (single-photon emission computerized tomography). She had an excellent response to immunotherapy [23].

In another case report, an adult man presented with persistent dystonic seizure of both upper limbs with T2/FLAIR, diffusion-weighted image hyperintensity in both hemispheres. He also responded well to immunotherapy [24].

A recent meta-analysis showed that around 40% of patients had an abnormal MRI (magnetic resonance imaging) in the acute phase. The most reported abnormalities were T2/FLAIR (Fluid-attenuated inversion recovery) hyperintensities often with DWI (Diffusion Weighted Image) restrictions in the temporal lobe, followed by cortical grey matter, and subcortical white matter [25].

#### 2. LGI1 Associated Encephalitis

The classical and pathognomonic movement disorder of LGI1 encephalitis is Facio-brachial dystonic seizure (FBDS). FBDS usually precede the onset of cognitive decline in half of the patients [26,27]. Although it is classified as seizure, usually there are no electrophysiological correlates of these episodes. Most of the studies report only movement artifacts during the episodes [28]. Dalmau et al. reported three patients with similar tonic seizures with preceding electro-decremental responses in EEG, suggesting an epileptic origin [29]. However, no study till now have shown conclusive ictal or interictal patterns.

The episodes usually last for less than 30 seconds and occur at a very high frequency of 5–10 per hour. Patients experience sudden, brief tonic contractions of the upper limb and one side of the face, and in rare instances, involvement of the leg also. Different phenotypes like tonic- dystonic, myoclonic- dystonic have been described [30,31]. Involvement of the limbs could be unilateral or alternating. Previously thought to have originated from basal ganglia [32] but a recent MEG-based (Magnetoelectrography) study showed clustering of dipoles around their origin in the insula coinciding with the most common area of MRI abnormality [33]. They usually do not respond to anti-seizure medications indicating towards non-epileptic nature, but they respond quite well to immunotherapies.

#### 3. Anti Ri associated encephalitis

Anti-Ri (also known as anti-neuronal antibody type 2, ANNA-2) antibody-associated encephalitis syndrome presents with a subacute onset cerebellar syndrome, opsoclonus- myoclonus ataxia syndrome or progressive brainstem syndrome (ophthalmoparesis, facial palsy) [34].

However, it can also present with dystonia involving cranial musculature, like cervical dystonia, oromandibular dystonia or blepharospasm. The prevalence of dystonia at presentation in a large series was 17% [35].

Oromandibular Dystonia seen in this syndrome was predominantly jaw-closing dystonia, causing intense spasms of jaw musculature interfering with mouth opening and feeding. This leads to complications like recurrent tongue bites, oral ulcers, recurrent temporomandibular joint dislocation, weight loss, laryngospasm, and even death. In an electromyography-based study, it was shown that these patients have co-contraction of both the masseter and lateral pterygoid simultaneously during voluntary jaw opening but not during jaw closure [36]. Immunotherapy is warranted, but many patients will have satisfactory responses to symptomatic treatment (anticholinergics, clonazepam) [37,38].

Episodic laryngospasm was observed, leading to spasmodic dysphonia or, in severe cases, stridor and respiratory distress requiring tracheostomy or ventilation [38]. Exact localization is still unclear but postulated to be due to over-excitation or disinhibition of the pontine tegmentum [39].

Cervical dystonia reported in anti-Ri Encephalitis is accompanied by oromandibular dystonia and other florid brainstem signs [40].

Blepharospasm was seen in a few cases, and the EMG study showed co-contraction of frontalis and orbicularis oculi muscles both at rest and during voluntary contraction [36].

The striking feature of dystonia in anti-Ri encephalitis syndrome was that it almost exclusively occurred in females or males with breast cancer. We hypothesize that it could be due to the presence of an onconeuronal antigen in breast tissue triggering cytotoxic CD8 cells to produce antibodies that target pontine tegmentum neurons.

#### 4. Anti IgLon5 (Immunoglobulin-like cell adhesion molecule 5) associated encephalitis

Anti IgLON5 disease is a recently described autoimmune disorder that connects the dots between neuroimmune and neurodegenerative pathology. The core clinical phenotypes are prominent sleep disturbances, bulbar symptoms, chorea, PSP (Progressive Supranuclear Palsy) phenotype, cognitive decline, and neuromuscular disorders. Among movement disorders, dystonia is the third commonest after gait disturbance (bradykinetic PSP-like gait) and chorea.

In a large series, 26% of patients presented with dystonia, and among them, 80% presented with craniocervical dystonia, including blepharospasm, cervical dystonia, lingual dystonia, and jaw-opening dystonia [41]. Many patients had painful dystonia, including trismus and painful cervical dystonia, which is uncommon in degenerative dystonias. Laryngospasm, along with craniocervical dystonia requiring ventilatory support, was also described [42]. Cranial preference was not unique to dystonia and was noticed with other movement disorders like fascio-lingual myoclonus [43].

Limb dystonia was subtle primarily, involving upper limbs, and rarely a presenting symptom [44] and was associated with dystonic tremors [45]. Few patients developed truncal dystonia with prominent antecollis and laterocollis resembling Parkinsonian pisa syndrome.

Patients with craniocervical dystonia had more response to symptomatic therapies like botulinum toxin injection and dopamine depleters like tetrabenazine and less response to immunotherapy. Those with limb dystonia had a better response to systemic immunotherapy [44].

#### 5. Anti GAD65 (Glutamic acid decarboxylase 65- kilodalton isoform) antibody-associated encephalitis

The spectrum of core clinical manifestations of this disease are epilepsy, stiff person syndrome, cerebellar ataxia, limbic encephalitis, and cognitive decline [46]. Dystonia described in these patients had mostly two patterns- 1) Insidious onset lower limb dystonia occurring more in the older population (5^th^–6^th^ decade) [47] and 2) Subacute onset craniocervical dystonia in middle-aged patients [48,49].

Limb dystonia was associated with hemiataxia, other hyperkinetic movement disorders like chorea, myoclonus [48,50] or ocular motor abnormality [51,52] Dystonia which affects younger adults with significantly high titre usually has a good response to immunotherapy [46]. Older age of onset with low titre of GAD65 and associated ataxia usually predicts poor response to immunotherapy and bad prognosis [46]. In a large series of 44 patients, two patients had dystonia. Among them, one had focal posture-sensitive limb dystonia with florid brainstem signs and another patient had blepharospasm [53]. Sometimes it is difficult to distinguish between focal limb dystonia with focal limb stiffness. Electromyography with a trial of intravenous benzodiazepine (diazepam) is a good tool to differentiate as dystonia will show no improvement but stiff person syndrome will show marked relaxation of the agonist and antagonist muscle [54,55,56].

#### 6. Anti GABA_A_ receptor Encephalitis

Anti GABA_A_ receptor encephalitis is an extremely rare form of autoimmune encephalitis which usually presents as refractory seizures, progressive cognitive decline in children often precipitated by infections, autoimmune conditions, or vaccination [57,58]. Movement disorders are the fourth most common symptom occurring in 14% of patients [59]. Among various hyperkinetic movement disorders choreiform facial dyskinesia was the commonest (43%) followed by ataxia. The phenomenology of most of the patients was mixed hyperkinetic with predominant orofaciolingual involvement like NMDA encephalitis and in some case reports it could not be exactly categorized into a specific movement disorder [60]. dystonia was never the presenting symptom in children and they occur along with a refractory seizure or prolonged encephalopathic state [61,60]. They have a good response to immunotherapy however often have a relapsing course [62,59].

#### 7. Anti D2R Encephalitis

Basal Ganglia encephalitis is a rare relapsing autoimmune encephalitis characterized by inflammation of basal ganglia and other deep grey matter nuclei. It occurs following infection (β-hemolytic streptococcus, mycoplasma, and enterovirus), post-inflammatory, post-vaccination period, and often in patients of systemic autoimmune disease. Dale et al divided it into two groups- one with anti-D2R positive, the second with anti-D2R negative [63]. The exact pathophysiological role of this antibody is still under review [64,65].

Patients were mainly divided into four phenotypes- 1. Basal Ganglia Encephalitis, 2. Tourette syndrome (TS), 3. Isolated psychosis (IP) and 4. Autoimmune Encephalitis (AE) [66]. Clinical phenotype is dominated by different movement disorders like dystonia, chorea, tics, parkinsonism, psychiatric symptoms (personality change, agitation), sleep disturbance (somnolence, insomnia), and rarely seizure. [67,68,69]. We have only considered seropositive patients in this review. It affects children and early adolescents and most often has a rapid onset following a trigger. Dystonia is usually limb onset focal dystonia or hemidystonia with coarse dystonic tremor [63]. Dystonia, particularly isolated focal limb dystonia progressing into hemidystonia was described as a prominent movement disorder in 75% (3/4) patients in a case series. They often progress to status dystonicus [70].

Oromandibular Dystonia including paroxysmal jaw clonus, blepharospasm, retrocolis, and postural limb tremor was also described in two adolescent female patients [71,69].

MRI reveals T2 hyperintensity in bilateral basal ganglia with edema and often bilateral striatal necrosis. FDG PET shows hypermetabolism of the affected region. On long-term follow up there is often progressive atrophy and cavitation of the affected areas [66,72]. No imaging abnormalities were found in patients with IP and TS groups [66].

They all had good responses to immunotherapy.

#### 8. Anti PDE 10 A (phosphodiesterase 10 A) Encephalitis

Recently Zekeridou et al [73] described a series of seven patients with phenotype of hyperkinetic movement disorders and encephalopathy. Among the hyperkinetic movement disorders chorea, ballismus, and generalized dystonia were described in three patients. In 2 patients MRI showed T2/FLAIR hyperintensity in bilateral basal ganglia. The clinical phenotype and MRI features closely resemble children with PDE 10 A mutation. It is also possible that this antibody is merely a marker for underlying cancer and not pathogenic.

#### 9. Anti Ma2 Encephalitis

Anti-Ma2 Encephalitis commonly presents with meso-diencephalic, limbic, or rhombencephalitis with or without MRI changes. It has a high association with tumors, especially testicular tumors. Movement disorders in this encephalitis are quite rare. Ataxia and eye movement abnormalities can occur due to rhombencephalitis, and isolated chorea is described in one case report. Another case report describes jaw-opening dystonia with parkinsonism in a 26-year-old male with a positive anti-Ma2 antibody [74].

In another case report a 70-year-old male presented with rapidly progressive symmetric akinetic rigid parkinsonism, left upper limb dystonic posturing, and orthostatic hypotension. His cranial and spinal MRI and cerebrospinal fluid test were unremarkable. However, he had Anti Ma2 antibody positivity in serum. He was treated with high-dose methylprednisolone resulting in improvement in his symptoms [75].

#### 10. Anti CV2/CRMP5 (Collapsin Response Mediator Protein 5) antibody-associated paraneoplastic encephalitis

Anti-CV2/collapsin response mediator protein 5 (CRMP5) associated encephalitis is a relatively rare form of paraneoplastic neurological symptoms. It often manifests as limbic encephalitis, chorea, ocular manifestation, cerebellar ataxia, myelopathy, and peripheral neuropathy. Apart from chorea other hyperkinetic movement disorders like dystonia, and ataxia are also described.

In a case series, Vermino et al. described 16 patients who presented with chorea, and 4 of them had dystonic posturing. All of them had combined dystonia, mostly limited to the limbs affected by chorea [76].

In another case report, Waheed et al. described another 57-year-old male patient with lung cancer who presented with small fiber neuropathy, chorea, and dystonia. Interestingly, he had dual positivity for both CRMP5 antibody and PCA-2 antibody [77].

Treatment is often aggressive, with early tumor search and removal, systemic immunosuppressants, and anti-tumor therapy. The response rate is not very encouraging; still, aggressive early institution of anti-tumor and immunosuppressants is necessary [78].

#### 11. Anti Myelin oligodendrocyte glycoprotein antibody disease (MOGAD)

Anti Myelin oligodendrocyte glycoprotein antibody-associated disease (MOGAD) is a childhood encephalitis presenting with recurrent optic neuritis, myelitis or encephalomyelitis, brainstem syndrome, or cerebral cortical encephalitis syndrome. The presentation of dystonia is rare compared to the other core classical features.

All the case reports describe limb dystonia or hemidystonia affecting both upper and lower limbs and orofacial dystonia in one child. All of them had abnormal electroencephalogram (EEG), and in two of them, dystonia was preceded by seizure, and they were on anti-seizure medications. One patient was an adult (at 28 years of age) [79], and the other two cases were described in children [80,81]. Although there were no electrophysiological correlates of the dystonic movements in the EEG, response to anti-seizure medication can suggest an underlying epileptic etiology; however, we need more data to ascertain this hypothesis. MRI brain in affected patients showed multifocal T2/FLAIR hyperintensity, and CSF findings were also compatible with MOGAD. All of them had an excellent response to immunotherapy. However, in severe cases, they required symptomatic Botulinum toxin and muscle relaxants.

#### 12. Anti-metabotropic glutamate receptor 5 encephalitis (Anti mGluR5 Encephalitis)

Anti-metabotropic glutamate receptor five encephalitis (Anti mGluR5 Encephalitis) is associated with neurocognitive symptom complex comprising reduced sensorium, mood disturbances, hallucination sleep disorder, seizures, dysautonomia, and classical ‘Ophelia syndrome’ associated with Hodgkin’s lymphoma.

In a large cohort of 29 patients, around 10% of patients (3/29) had dystonia [82].

Among the case reports where dystonia is a presenting feature, one patient is from the pediatric age group (Female,6 years) [83] who also developed an oculogyric crisis, and others are adults. All of them had multiaxial symptoms with prominent cognitive dysfunction, seizure, and sleep disturbances. Two of them (2/4) had an association with Hodgkin’s Lymphoma, one patient had associated mature teratoma, and another patient did not have any evidence of cancer. All of them had generalized dystonia with predominant orofacial dystonia [82,84]. Treatment response was encouraging with combined immunotherapy and anti-tumor therapy. All patients underwent treatment for underlying cancer, and additionally, 85% required first-line immunotherapy, 20% required second-line immunotherapy, and 15% had clinical relapse despite the treatment [85].

### B. Dystonia caused by systemic autoimmune diseases

Autoimmune disorders have multisystemic involvement due to widespread immune complex deposition and antibody-mediated cytotoxicity. They are caused by loss of self-tolerance and production of autoantibodies, which induces inflammatory changes in the target organs, including the central and peripheral nervous systems. Recently, after the recognition of distinct movement disorders related to pathogenic neuronal antibodies, it was observed that patients also have a distinguishable spectrum of movement disorders in association with systemic autoantibodies. This review describes the patterns of dystonia in different systemic autoimmune diseases.

#### 1. Sjogren Syndrome

In a case series of patients with seronegative Sjogren’s with positive minor salivary gland biopsy dystonia was observed as a rare manifestation, only in 2.2% of patients [86]. In a review Menozzi et al have described two phenotypes of dystonia [87].

First, slowly progressive subacute focal or segmental dystonia affecting the craniocervical region in older females with a normal brain imaging and good response to immunotherapy and botulinum toxin administration [88,89,90,91]. The distribution of dystonia was oromandibular dystonia in three patients; among them, 2 also had blepharospasm, and one had spasmodic dysphonia. Another two patients had cervical dystonia.

Second, paroxysmal (less than 2 minutes), painful dystonic spasms of one or both upper or lower limbs in younger females triggered by sudden movements. They usually have T2/FLAIR signal changes in the brainstem or spinal cord and have mixed responses to immunotherapy and sodium channel blockers. As Sjogren syndrome shares a close overlap with NMOSD (Neuromyelitis Optica Spectrum Disorder), we cannot rule out NMOSD in these cases as the specific AQP antibodies (Aquaporin antibody) were not tested in these patients [92,93,94]. However, in one case, CSF AQP-4 antibody was negative, and Anti Ro antibody was positive in high titre with MRI showing hyperintensity in the cervical cord [95].

#### 2. Systemic Lupus Erythematosus (SLE)

The most common movement disorder in patients with SLE is chorea. There are a few case reports of dystonia in patients of SLE with two clinical patterns- One with contributory MRI lesion (MR positive) and one without any corroborative MRI lesion (MR negative). In MR-negative patients, dystonia is caused by direct cytotoxicity of the antibodies associated with SLE.

MRI-positive patients present with a complex movement disorder including combined dystonia, parkinsonism, choreoathetoid movements [96,97,98,99]. They are more common in middle age (range 18–40 years) males. One patient with hemidystonia–hemiparkinsonism showed contralateral basal ganglia T2/FLAIR signal changes [100]. Other patients with hyperkinetic movement disorders had bilateral symmetrical basal ganglia changes. One patient with complex hyperkinetic myoclonus- Dystonia- choreoathetoid showed multiple cortical-subcortical FLAIR hyperintensities [98]. Another female with acute facial dystonia with speech difficulty also showed symmetrical basal ganglia changes [97]. All the patients had a very good response to initial steroid treatment and remained in remission with continuous immunosuppressants.

Patients with MRI-negative dystonia were all female at the extremes of age (range: 9–56 years). They usually present with isolated focal or hemidystonia [101,102,103,104]. All of them had ANA positive with 75% (3/4) having a speckled pattern. Anti-ds DNA was done in three patients, and it was positive in all cases. Compliment factors C3, and C4 levels were normal in all patients indicating the pathogenesis was not due to immune complex deposition but direct antibody-mediated cytotoxicity. The response to steroids was mixed. One young child with writer’s cramp had a very good response to steroids [101,96]. Another 31-year-old female with right foot dystonia was and required botulinum toxin injection [102].

#### 3. Anti Phospholipid Antibody Syndrome (APS)

Movement disorder is a rare neurological presentation of APS [105].

Among the limited data available total of eight case reports were included and none of the patients had isolated dystonia with all of them having corroborative lesions on MRI [106,107,108,109,110,111]. The age range was between 7 years to 76 years with female predominance (4/6). The dystonia observed were focal limbdystonia (2/8), hemidystonia (4/8), oromandibular dystonia (2/8).

Three children had hemidystonia with or without chorea and corresponding MRI signal changes. All of them had positive anticardiolipin antibody in high titre [108].

Another 60 year woman with writer’s cramp progressed to right hemidystonia with parkinsonism [106]. In another case report a 51-year-old woman presented with ataxia, parkinsonism with generalized chorea, and Dystonia with MRI lesion. Immunotherapy reduced chorea and dystonia but not parkinsonism.

Although chorea has a well-established immunopathological background in APS the same is not the case with dystonia [112]. Whether it holds a temporal association or just an epiphenomenon of this antibody is yet to be determined. While some patients with dystonia show robust response to immunotherapy similar to those in chorea [109], others do not respond to immunotherapy [106,108].

#### 4. Neuro Behcets Disease (NBD)

Neurological manifestations of Behcet’s disease are traditionally classified as parenchymal NBD or non-parenchymal NBD. Parenchymal NBD includes signs of the brainstem, basal ganglia, and cerebral or spinal cord involvement [113]. Despite common basal ganglia involvement in MRI, movement disorders are rare in patients with Behcet’s disease. In a large case series 6% of patients had movement disorders [114]. It involves young males between 2^nd^–3^rd^ decades. The most common presentation is paroxysmal focal dystonia or hemidystonia with corresponding MRI lesions in basal ganglia with or without brain stem and cerebral cortex [115,116,117].

Patients usually have good response to immunosuppressives and D2 blocker Haloperidol, with one patient treated with carbamazepine because of epileptiform discharges on EEG [117].

## Discussion

This scoping review gives an overview of the existing literature on the patterns of dystonia in different autoimmune disorders **(Supplemental Table–1)**. The temporal association of dystonia with an autoimmune disorder could be evident from the short history, associated clinical features, abnormal brain imaging, and serological tests. In this situation, the pattern of dystonia often helps to elucidate the antibody association in the proper clinical context.

**Figure 2:**
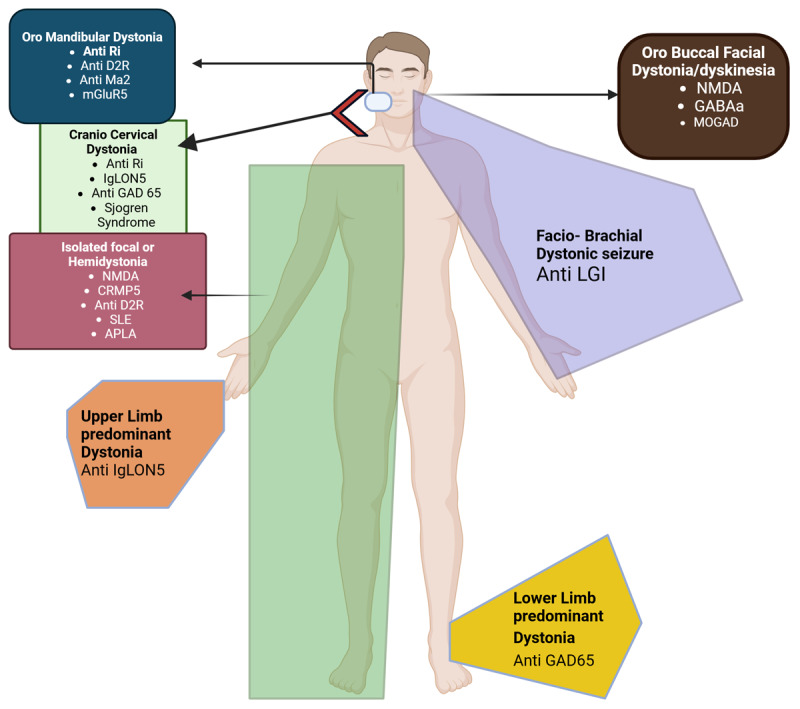
Clinical pattern of dystonia associated with autoimmune disorder.

**Figure 3:**
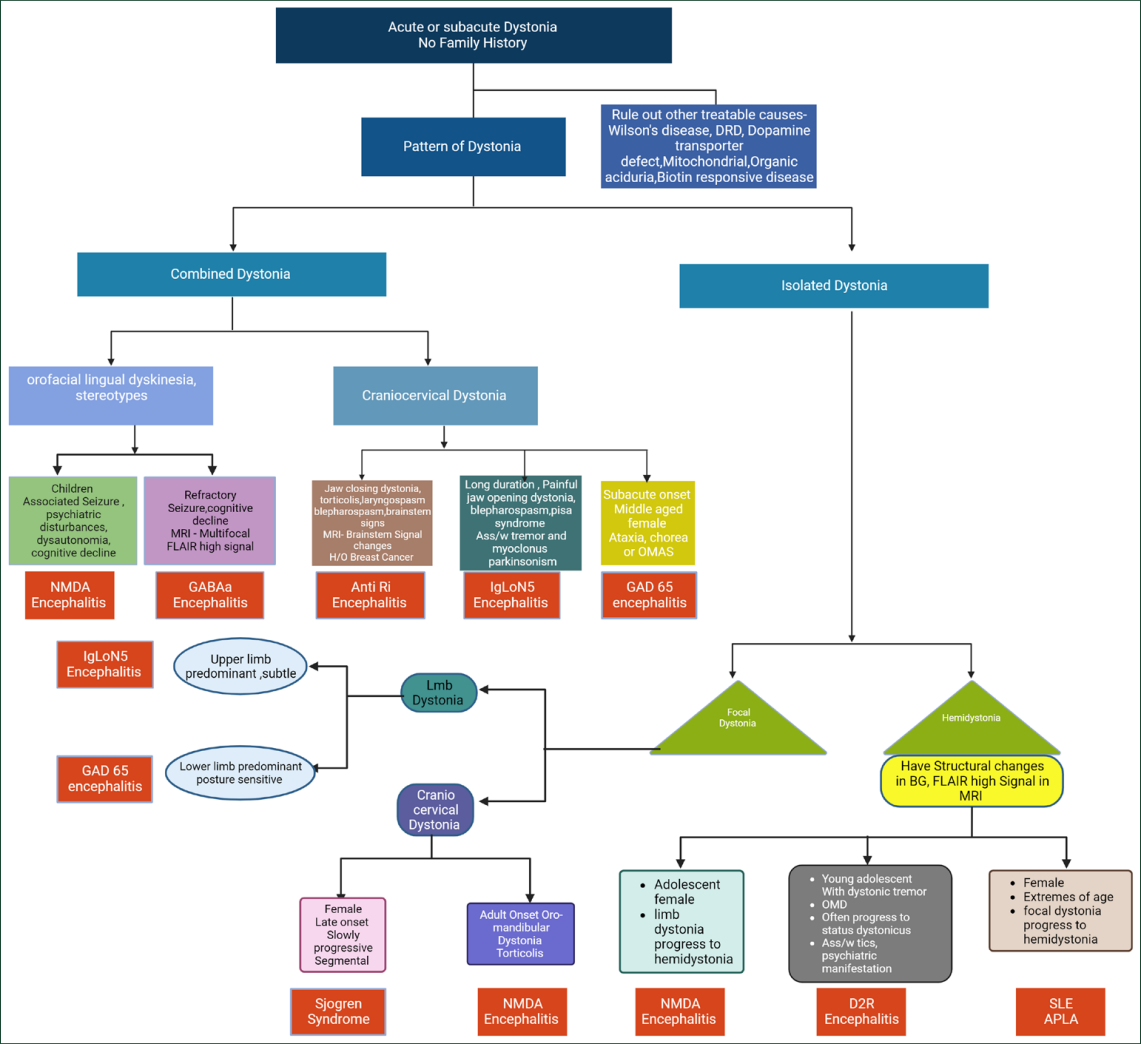
Clinical approach to dystonia in autoimmune disorders.

In this review, we propose that there is a phenotypic distinction between dystonia occurring as part of the classical phenotype and dystonia appearing as an isolated manifestation of an autoimmune disorder. In the latter situation, it is difficult to diagnose, often even suspect an underlying autoimmune condition. In this article, we suggest a clinical algorithm and describe a pattern recognition of acute to subacute onset dystonia in the context of various systemic and CNS autoimmune diseases (Figures 2 and 3). Recognizing these potentially treatable disorders is important because early diagnosis often confers a good long-term prognosis.

The pathophysiological substrate of dystonia, generalized for all types of dystonia, is related to three lines of research. The first postulate is disinhibition which may account for the excess of movement and for the overflow phenomena. A second abnormality is a lack of sensory-motor integration, which is related to mild sensory complaints in patients with focal dystonias and may be responsible for some of the motor dysfunction. Finally, there are strong pieces of evidence from animal and human studies suggesting that alterations of synaptic plasticity characterized by a maladaptive homeostatic plasticity, with a prevailing facilitation of synaptic potentiation may play a pivotal role in primary dystonia [118].

The association between dystonia and autoimmune disorders has been recognized for a long time but the pathomechanism is far from being understood. In some cases, there is a direct pathological link between the antibody and specific movement disorders like antiphospholipid antibodies and chorea but that is not the case in most autoimmune dystonia.

The relationship between an autoimmune etiology and dystonia is most evident in cervical dystonia. There are anecdotal reports linking cervical dystonia with autoimmune thyroid disease [119]. In an exploratory study, authors have shown that there is a significant (p < 0.5) difference between different proteins like dopamine-β-hydroxylase (DBH), factor XIIIA1 (F13 A1), and hepatocyte growth factor activator (HGFAC) and other proteins related to acute inflammatory response, acute phase response in patients with cervical dystonia with or without thyroid disease compared to normal control population. The same study also showed an increased population of B cells, monocytes, and an increased ratio of CD8+ cytotoxic cells compared to CD4+ helper cells with an increase in proinflammatory cytokines like IL-5, IL-2, PIGF, VEGF-D, IL-1ß in patients of cervical dystonia. All of which indicates an altered immunological profile associated with cervical dystonia [120].

The understanding that autoantibodies can contribute to neurological dysfunction has brought about a paradigm shift over the past decade. Detection of specific autoantibodies to neuronal or glial targets has resulted in a better understanding of the central nervous system autoimmunity and reclassification of some ‘idiopathic’ or ‘psychogenic’ entities. One of the glaring examples of this is NMDAR antibody-associated encephalitis. The pathophysiology of NMDAR encephalitis has been well described in the literature. However, the hyperkinetic movement disorders, including dystonia/dyskinesia in this encephalitis is postulated to be caused by the disruption surface interaction between EPHB2R (Ephrin-B2 receptors) and GluN2 A subunits of NMDAR by the IgG antibodies against NMDAR causing lateral dispersal of synaptic EPHB2R and NMDAR [121]. NMDA helps in long-term potentiation and synaptic plasticity of medium spiny neurons in the striatum helping in coordinated learned motor behavior [122]. With dysfunctional NMDAR there is a significant increase in the magnitude of LTP coupled with a loss of both LTD (Long-term depression) and SD (Synaptic Depotentiation) which are essential cellular mechanisms to erase unnecessary or redundant information, leading to maladaptive plasticity and dystonia [123].

In Anti Ri encephalitis, IgG binds to an intracellular RNA binding protein named NOVA-1 and NOVA-2, which are expressed in the ventral brainstem and spinal cord, causing neuronal death by CD8+ T Cells, and it is thought to be the main pathological substrate for jaw dystonia and laryngospasm seen specifically in this condition [124,125,38]. In another autoimmune encephalitis (Anti GABAa, anti-GAD65), there is antibody-mediated blockade of GABA transmission, which leads to decreased intrastriatal inhibitory signals causing dystonia and other hyperkinetic movement disorders [126].

In Basal ganglia encephalitis, the presence of IgG antibody against the extracellular domain of postsynaptic surface D2 long receptor is postulated as pathological. The D2 receptor is essentially an inhibitory receptor. Anti-D2 receptor IgG antibody binds with neurons in the striatum and blocks the inhibitory D2 receptor through activation of CaMKII (calcium calmodulin-dependent protein kinase II) leading to excess dopamine release causing hyperkinetic movement disorders [63,127].

In anti IgLON5 Encephalitis, there is in vivo evidence of the accumulation of p-Tau and death of nigrostriatal dopaminergic neurons leading to persistent motor impairment [128].

The cooccurrence of two autoimmune diseases could be another explanation for some of the dystonia, like paroxysmal Dystonia in Sjogren syndrome. The dystonia likely represents painful tonic spasms in Neuromyelitis Optica with AQP4 antibodies, as the association between these two diseases is very strong [129]. These antibody-mediated dysfunction of dopamine, GABA, and NMDA receptors either directly or indirectly cause decreased inhibitory signals within the basal ganglia circuitry, leading to dystonia and other hyperkinetic movement disorders.

A large number of patients with acute or subacute onset dystonia without any family history remain idiopathic even after extensive investigation. A particular pattern of dystonia in these cases might point towards an autoimmune origin. Like an isolated painful craniocervical dystonia with dystonic tremor or myoclonus of the same region with subtle upper limb dystonia may point towards IgLon5 encephalitis [43,41]. Similarly, isolated lower limb dystonia with stiffness or axial dystonia may indicate GAD65-associated dystonia [47] or jaw-closing dystonia with torticollis, blepharospasm, or laryngospasm may indicate Anti Ri encephalitis-associated dystonia [39,36]. In the same way, a single form of encephalitis can present with various phenotypes like NMDAR encephalitis presents as orofacial dyskinesia, stereotypies with seizure cognitive decline in children and adolescents, but in adults, they present with isolated limb dystonia progressing to hemidystonia or oromandibular dystonia with torticollis [17,15]. Similarly, paroxysmal dystonic spasms in middle-aged females or late-onset craniocervical dystonia may indicate Sjogren syndrome or isolated unilateral focal or limb dystonia may indicate antiphospholipid syndrome.

Neuroimaging features in most autoimmune disorders are diverse and potentially can involve any region of the brain. Dystonic presentation in central nervous system autoimmune diseases usually has the same typical imaging features of the particular antibody-associated encephalitis. Some of the MRI changes are non-specific and could not explain the causal relationship between the brain lesion and dystonia like in NMDA (bitemporal hyperintensity and orofacial dyskinesia), LGI, IgLoN5 (cerebral atrophy and limb dystonia), GAD65 (cerebral atrophy and stiff person syndrome or lower limb dystonia), GABAA, MOG, mGluR5 associated encephalitis. However, in some CNS autoimmune diseases, the MRI signal changes could explain the anatomical localization of Dystonia like Anti Ri (brainstem signal changes and jaw dystonia and laryngospasm), D2R antibody (Bilateral basal ganglia signal changes and dystonia), Ma2 antibody (brainstem signal changes and jaw dystonia or upper limb dystonia). In systemic autoimmune diseases, the pattern is mixed. There can be corroborative changes in MRI where the MR lesion can directly explain the anatomical localization of dystonia (like ischemic or demyelinating changes in basal ganglia in SLE or APS and contralateral hemidystonia) or paroxysmal dystonic attacks and cervical cord signal changes in Sjogren Syndrome. In some cases the MRI changes can be non-specific and cannot explain the anatomic localization of dystonia.

Treatment response to dystonia in autoimmune disorders also follows the conventional pattern. Focal dystonia like blepharospasm and oromandibular dystonia usually have moderate to good responses to symptomatic therapy, including anticholinergics, centrally acting muscle relaxants (Baclofen), and specifically botulinum toxin. Antibodies against neuronal surface antigens (like NMDA, LGI, D2R, GABAA, MOGAD, and mGluR5) have a good response to immunotherapy. They are rarely associated with malignancy, but when they are, cancer treatment becomes necessary. On the contrary paraneoplastic encephalitis with antibodies against intracellular cytoplasmic/nuclear antigens (like Anti Ri, PDE10 A, Ma2, CRMP5) has a poor response to immunotherapy with or without cancer treatment [130]. However, there are some exceptions, like in anti IgLoN5 encephalitis limb dystonia responds very well to immunotherapy but craniocervical dystonia with myoclonus responds better with local symptomatic therapy [43,44], in GAD-65 associated encephalitis young patients with high titre tend to have a better response with immunotherapy, and older patients with more atrophy have a poorer response [46,55]. Similarly, in Sjogren syndrome, craniocervical dystonia has a better response to immunotherapy than paroxysmal dystonic reaction [88,89]. In SLE, those with symptomatic MRI lesions respond better to immunotherapy than those with non-specific MRI changes.

Main Limitation of this scoping review is the heterogeneity of the studies included, small sample size. Since dystonia is a rare presentation there is always a high risk of bias which was not assessed in scoping reviews. It also does not assess the quality of the studies included hence the review is broad at the expense of its depth.

## Conclusion

In summary, whether the presence of autoantibodies in subacute dystonia is an epiphenomenon or holds an actual temporal correlation is far from being understood. This review demonstrates that the clinical pattern of dystonia may be unique depending on the underlying antibody association (Table 2). This review will provide the building blocks for conducting longitudinal studies to ascertain possible disease associations described herein. If proven, this may change our approach towards acute and subacute onset dystonia and open new avenues for treatment.

**Table 2:** Search Strategy in Pubmed.


		AND		OR	

*DATABASE*	*SEARCH TERM 1*		*SEARCH TERM 2*		*SEARCH TERM 3*

**Pubmed**	**NMDA Encephalitis**		Dystonia/Dyskinesia		Movement Disorder

	**LGI1 Associated Encephalitis**		Dystonia/Dystonic seizure		Movement Disorder

	**Anti Ri associated encephalitis**		Dystonia		Movement Disorder

	**Anti IgLon5 associated encephalitis:**		Dystonia		Movement Disorder

	**Anti GAD65 antibody associated Encephalitis:**		Dystonia		Movement Disorder

	**Anti GABA receptor Encephalitis:**		Dystonia/Dyskinesia		Movement Disorder

	**Anti D2R Encephalitis**		Dystonia		Movement Disorder

	**Anti PDE 10 A Encephalitis**		Dystonia		Movement Disorder

	**Anti Ma2 Encephalitis**		Dystonia		Movement Disorder

	**Anti CRMP5**		Dystonia		Movement Disorder

	**MOG Antibody Disease**		Dystonia		Movement Disorder

	**Anti mGluR5**		Dystonia		Movement Disorder

	**Sjogren Syndrome**		Dystonia		Movement Disorder

	**Systemic Lupus Erythematosus (SLE)**		Dystonia		Movement Disorder

	**Neurobehcet’s Disease**		Dystonia		Movement Disorder


## Data Accessibility Statement

The data that support the findings of this study are openly available in the public domain resources.

## Additional File

The additional file for this article can be found as follows:

10.5334/tohm.915.s1 Supplemental Table 1. Main characteristics of the studies on dystonia in different autoimmune disorders.

## Abbreviations

aCL = Anti cardiolipin antibody

Anti β2-GP1 = Anti β2 Glycoprotein 1

FBDS = Faciobrachial Dystonic Seizures

IT = Immunotherapy

IVIg = Intravenous Immunoglobulin

IVMPS = Intravenous Methyl Prednisolone

LE = Limbic Ebcephalitis

MD = Movement Disorder

MR = Magnetic Resonance

NMDA encephalitis = Anti-*N*-methyl-d-aspartate receptor encephalitis

OMAS = Opsoclonus-Myoclonus Ataxia Syndrome

OMD = Oro Mandibular Dystonia

PLEX = Plasma Exchange

SPS = Stiff Person Syndrome

TS = Tourette Syndrome
