# Supplementary material for: Decoding Dystonia in Autoimmune Disorders: A Scoping Review
Source: Tremor Other Hyperkinet Mov (N Y). 2024 Dec 6;14:60. doi: 10.5334/tohm.915 (PMC11623079; doi:10.5334/tohm.915)
Supplement: Supplemental Table 1. — Main characteristics of the studies on dystonia in different autoimmune disorders. [file tohm-14-1-915-s1.pdf]

SUPPLEMENTAL MATERIAL:

Supplemental Table 1 – Main characteristics of the studies on dystonia in different autoimmune disorders

	**Article title**	**Author**	**Study Design**	**Year of publication**	**Sample Size**	**Antibody/Disease association**	**Summary of study**
1.	Treatment and prognostic factors for long-term outcome in patients with anti-NMDA receptor encephalitis: an observational cohort study	Maarten J Titulaer et al	Observational cohort study	2012	577	NMDA	55 children (50%) younger than 12 years presented with seizures or movement disorders(orofacial dyskinesia and choreoathetosis). The occurrence of abnormal behavior, movement disorders, and seizures was different between the three age groups (p<0·0001; Figure 2) Within the first 4 weeks of symptom onset, most patients developed a similar spectrum of symptoms irrespective of their age. Although movement disorders were more common in children, memory deficits and central hypoventilation occurred more often in adults. 94% underwent first-line immunotherapy or tumor removal, resulting in improvement within 4 weeks in 53%.
2.	Variations of movement disorders in anti-N-	Bi-Chun Duan et al	Retrospective Observational	2016	28	NMDA	OFLD was second most prevalent movement disorder in children less than 18 years (second only

	methyl-D-aspartate receptor encephalitis A nationwide study in Taiwan						to choreoathetosis and catatonia) and the most common movement disorder in patients more than 18 years
3.	Anti-N-Methyl d Aspartate Receptor Encephalitis	Nidhiben Anadani, MD	Literature review	2020		NMDA	Dystonia, chorea, and stereotypies were among the most common movement disorders, and 97% and 76% of participants had 2 or 3 of these, respectively
4.	The Distinctive Movement Disorder of Ovarian Teratoma-Associated Encephalitis	Timothy J. Kleinig, FRACP, Philip D. Thompson, PhD et al	Case Series	2008	4	NMDA	The characteristics of the movement disorder differed from recognized dyskinesias.All (4/4) had orofacial complex movements , one (1/4) had limb dystonia, dystonic hand posturing.
5.	The Spectrum of Movement Disorders in Anti-N-Methyl-D-Aspartate Receptor (NMDAR) Encephalitis Both in Children and Adults: An Experience From a Single Tertiary Care Center	Ayaz Ul Haq et al	Retrospective observational	2021	17	NMDA	orofacial lingual dyskinesia being the most common movement disorder in all pediatric patients with NMDA receptor encephalitis
6.	The Movement disorder associated with NMDAR antibody-encephalitis is complex and characteristic: An expert video-rating study	Varley, James A et al	Letter to editor	2019	34	NMDA	The unusual triad of dystonia, stereotypies and chorea, should, in the correct clinical context, provide clinicians with greater confidence in diagnosing this common cause of encephalitis, allowing earlier immunotherapy administration and improved patient outcomes.
7.	Severe childhood encephalopath	Poloni, Claudia Et al	Case Series	2010	4	NMDA	Author described unilateral foot dystonia which occurred well before the

	y with dyskinesia and prolonged cognitive disturbances: evidence for anti-N-methyl-D-aspartate receptor encephalitis						onset of constellation of symptoms (severe encephalopathy with massive hyperkinesia, marked neurological and cognitive regression, sleep disturbance, prolonged mutism, and a remarkably delayed recovery (time to full recovery between 5 and 18mo).
8.	Isolated Craniocervical Dystonia Without Initial Neuropsychiatri ri Manifestation s Associated with NMDA-Receptor Antibodies	Waller, Sophie E, Fung, Victor S C et al	Case Report	2023	1	NMDA	35 year female with 2 month history of blepharospasm and craniocervical dystonia. MRI and EEG was normal. Serum and CSF NMDA was positive. She was started on IV steroids and IVIg followed by Rituximab. She had very good response to immunotherapy and developed mild psychiatric and cognitive symptoms on follow up.
9.	Progressive striatal necrosis associated with anti-NMDA receptor antibodies	Tzoulis C, et al.	Case Report	2013	1	NMDA	Isolated progressive generalized dystonia with bilateral striatal necrosis having onset at the age of 9 years. He did not have any psychiatric, behavioral, cognitive dysfunction. MRI Brain showed involvement of dorsal striatum with corresponding hypometabolism in PET CT.CSF showed raised protein with NMDA positivity. Genetics and wilson's work up was negative.
10.	Isolated hemidystonia associated with NMDA receptor antibodies.	Ignacio R et al	Case Report	2011	1	NMDA	Young female with acute left hemidystonia of hand, foot and face without any sensory tricks or mirror movements with normal MRI with good response to systemic immunotherapy
11.	Movement disorders in children with anti-NMDAR encephalitis	Mohamma d SS, Fung VSC et al.	Video case series	2014	31	NMDA	Dystonia was the second most common movement disorder noted in this group . Three patients had craniocervical dystonia, two

	and other autoimmune encephalopathies.						patients had dystonic spasms, and one patient had oculogyric crises
12.	Pediatric NMDAR encephalitis: A single center observation study with a closer look at movement disorders.	Granata T, Matricardi S et al.	Retrospective observational study	2018	18	NMDA	Distribution of dystonic posturing was variable with trunk dystonia with spontaneous opisthotonos posturing being the most common
13.	Antibody-related movement disorders – a comprehensive review of phenotypeautoantibody correlations and a guide to testing	Felix Göver et al	Literature Review	2020		NMDA	Distinct and complex hyperkinetic movements affecting the mouth and the limbs are a characteristic clinical feature of NMDAR encephalitis. Oromandibular and limb localization of dystonia is most common, although oculogyric crises are possible and rarely even generalized dystonia has been observed.
14.	Bilateral thalamic changes in anti-NMDAR encephalitis presenting with hemichorea and dystonia and acute transient psychotic disorder	Souvik Dubey et al	Case Report	2020	1	NMDA	A 34 year woman with right hemichorea and hemibody dystonia with psychiatric features had a good response to immunotherapy
15.	Anti-NMDA Receptor Encephalitis with a Favorable Prognosis Despite Delayed Treatment Due to Longstanding Psychiatric	Kim SW, et al	Case report	2014	1	NMDA	A female patient with visual and auditory hallucinations, upper limb dystonia ,oculogyric crisis , catatonia ,altered sensorium. MRI showing bilateral medial temporal FLAIR hyperintensity and EEG showed diffuse slowing. She responded well to immunotherapy.

	Symptoms						
16.	Dystonic Seizures and Intense Hyperperfusion of the Basal Ganglia in a Patient with Anti-N-Methyl-D-Aspartate Receptor Encephalitis.	Matsumoto H et al	Case Report	2017	1	NMDA	Young female with right dominant dystonic seizure including faciobrachial dystonic seizure with hyperactivity of ipsilateral insula and basal ganglia on SPECT secondary to NMDA encephalitis. She responded well to immunotherapy
17.	Anti-NMDAR encephalitis with bilateral basal ganglia MRI lesions at a distance of time: a case report	Son DK, et al	Case report	2022	1	NMDA	A 66 year man with bilateral tonic clonic and dystonic seizure and MRI lesion in both hemisphere. He responded well to immunotherapy
18.	Anti-NMDA Receptor Autoimmune Encephalitis: Diagnosis and Management Strategies.	Nguyen L, Wang C.	Systematic review	2023	1427	NMDA	Dystonia was second most common movement disorder. 40% patients had abnormal MRI.
19.	Initial clinical presentation of young children with N-methyl-D-aspartate receptor encephalitis	Marion Favier et al	Retrospective observational	2017	50	NMDA	Presentation of NMDA-R-Abs encephalitis in young children and different than in adults. The diagnosis should be considered in case of abnormal behavior, abnormal movements, recent seizures (focal or generalized) in the absence of obvious other etiology.One patient in this cohort had facio-buccal dystonia in evolution (Patient 10)
20.	Pediatric Anti-N-Methyl-D-Aspartate (NMDA) Receptor Encephalitis: Experience of a Tertiary Care Teaching Center From North India	Biswaroop Chakrabarty, DM et al	Retrospective observational	2013	11	NMDA	The common modes of presentation were progressive extrapyramidal syndrome with global neuroregression in 45% (5 of 11), epileptiform encephalopathy in 27% (3 of 11), and an overlap between the 2 in 27% (3 of 11). Extrapyramidal features included orofacial and limb dyskinesia

							in 64% (7 of 11), generalized dystonia in 27% (3 of 11), hemidystonia in 9% (1 of 11), and mutism in 36% (4 of 11)Fifty-eight percent showed significant response to steroids and IVIg.
21.	Clinical Features and Therapeutic Effects of Anti-leucine-rich Glioma Inactivated 1 Encephalitis: A Systematic Review.	Takeuchi H et al.	Systematic review	2022	485	LGI	Short-term memory loss (75.22%), faciobrachial dystonic seizures (FBDS) (52.53%), other seizures excluding FBDS (68.48%), psychiatric symptoms (57.67%), and sleep disturbances (34.30%) were the most frequently described symptoms in anti-LGI1 encephalitis.
22.	Clinical features of limbic encephalitis with LGI1 antibody.	Wang M, et al	Case series	2017	10	LGI	8 out of 10 patients had FBDS. Brain MRI shows mediotemporal lobe and the hippocampus involvement.
23.	Tonic seizures: A diagnostic clue of anti-LGI1 encephalitis?	Andrade DM, Tai P, Dalmau et al	Case series	2011	3	LGI	Though the motor movements were not typical of dyskinesias or myoclonus, in most instances ictal EEG recordings were unremarkable and, therefore, the movements could easily have been non-epileptic.
24.	. LGI1 Encephalitis: Autoimmune Epilepsy or Movement Disorder.	Jagtap SA, Aurangabadkar K, Joshi A, Chitnis S, Rathod M, Khade H	Case Series	2023	7	LGI	Long term Video EEG monitoring in patients with FBDS showed Five had alternating FBDS, some events involving the right side and some events involving the left side.Leg onset FBDS were present in three patients. The duration of FBDS was <15–20 s, occurring at a frequency of 5–10/h. EEG during FBDS showed only movement artifacts and no ictal pattern.
25.	Clinical features and long-term outcomes of	Qiao S et al.	Retrospective observational study	2021	117	LGI	The median age of all patients at the onset was 57 years. The median time from symptom onset to

	anti-leucine-rich glioma-inactivated 1 encephalitis: A multi-center study.						diagnosis was 8.7 weeks (IQR, 4.2–25). The main clinical features identified were seizures, cognitive impairment, and mental and behavioral abnormalities. Symptoms including memory, mental ability, and behavior improved after immunotherapy.
26.	Clinical characterization of autoimmune LGI1 antibody limbic encephalitis.	Gao L et al	Retrospective observational	2016	10	LGI	MEG study in two patients during the acute disease phase showed a small quantity of spike–wave dipoles in the temporal lobe close to the lateral fissure and insular lobe.
27.	Clinical spectrum and diagnostic pitfalls of neurologic syndromes with Ri antibodies. 2020;0:1–9.	Simard C et al	Retrospective observational	2020	36	Ri	At onset, 4 main patterns were observed: cerebellar syndrome (39%), isolated tremor (24%), oculomotor disturbances (17%), and other symptoms (19%). Patients manifested a variety of movement disorders, including myoclonus (33%), dystonia (17%), either cervical or oromandibular, and parkinsonism (17%). Most patients had cancer (92%), mainly breast cancer (n = 22). Survival at 12 months was 73% (95% CI [0.54–0.85]), at 24 months 62% (95% CI [0.41–0.78]), and at 36 months 47% (95% CI [0.25–0.65]).
28.	‘ Jaw clenching ’ in anti-Ri – Antibody-associated paraneoplastic syndrome.	Bekircan-kurt CE, Temucin ÇM, Elibol B, Saka E.	Letter to editor	2013	1	Ri	EMG of the masseter muscle revealed no activity at rest. Simultaneous EMG recordings of masseters, temporalis and lateral pterygoid muscles revealed co-contraction of agonists and antagonists with jaw opening but not with jaw closing. In addition, both at rest and during the voluntary closing of the eyelids, normal activity at the orbicularis oculi muscle was accompanied by abnormal

							activity of the frontalis muscles.<br>These co-contractions of antagonist muscles during the jaw and eye closing support the dystonic nature of this patient's involuntary movements
29.	Jaw Dystonia and Myelopathy : Paraneoplastic Manifestations of Breast Malignancy with anti - Ri / ANNA - 2 Antibody.	Tomar L et al	Letter to editor	2021	1	Ri	A 52 year female with progressive quadriparesis with jaw closing oromandibular dystonia in association with breast carcinoma. She had poor response to immunotherapy ,mild improvement to symptomatic therapy.
30.	Anti-Ri-Antibody-Associated Paraneoplastic Syndrome in a Man with Breast Cancer Showing a Reversible Pontine Lesion on MRI.	Kim H, Lim Y, Kim K.	Case Report	2009	1	Ri	A 52 year male patient with breast cancer presenting as complete horizontal ophthalmoplegia, left trigeminal sensory symptoms, and truncal ataxia. MRI showed a high-signal-intensity lesion in the pontine tegmentum. His treatment including intravenous immunoglobulin and oral prednisolone, symptomps reduced with reduced titre of antibody.
31.	Paraneoplastic Jaw Dystonia and Laryngospasm With Antineuronal Nuclear Autoantibody Type 2 (Anti-Ri).	Duncan PR et al	Retrospective case series	2016	48	Ri	Jaw dystonia and laryngospasm are common accompaniments ofANNA-2 autoimmunity and are associated with significant morbidity. We propose that selective damage to antigen-containing inhibitory fibers innervating bulbar motor nuclei by CD8 positive T lymphocytes (histopathologically observed infiltrating brainstem reticular formation) is the proximal cause of this syndrome. Early and aggressive therapy offers the prospect of neurologic improvement or stabilization.
32.	Subacute	Tisavipat N	Case	2023	1	Ri	A 61 year female with

	Horizontal Diplopia, Jaw Dystonia, and Laryngospasm.	et al.	report				Subacute onset Jaw dystonia and laryngospasm causing cyanosis. Jaw dystonia can also cause eating difficulty, resulting in severe weight loss and malnutrition. In this report, we highlight the multidisciplinary management of this syndrome.
33.	Anti-Ri-Associated Paraneoplastic Neurological Syndrome Revealing Breast Cancer :	Tazi R et al	Case Report	2022	1	Ri	60-year-old woman with a subacute static-kinetic cerebellar syndrome, cervical dystonia, and multiple cranial nerve palsies revealing a mammary adenocarcinoma with good response to immunotherapy.
34.	Bilateral horizontal gaze palsy due to Anti-IgLON5 disease.	Bhatti MT et al	Case report	2022	1	IgLON5	A 60 year female with oromandibular dystonia, mild hyperkinetic movement of the face and upper extremities, poor sleep and difficulty walking. Tried with Botox but had ill sustained response and hence started on IVIg.
35.	IgLON5 antibody. Neurol Neuroimmunol NeuroInflammation.	Honorat JA et al.	Retrospective case series	2017	20	IgLON5	Median disease-onset age was 62 years (range, 46–75 years). Clinical features included - Sleep disorders like sleep-disordered breathing (11) and parasomnias (3). Brainstem disorders were gait instability (14), dysphagia (10), abnormal eye movements (7), respiratory dysfunction (6), ataxia (5), craniocervical dystonia (3), and dysarthria (3). Seven of 9 immunotherapy-treated patients improved: 6 of those 7 were stable.
36.	Delayed Benefit From Aggressive Immunotherapy in Waxing and Waning Anti-IgLON5	Shambrook P et al.	Case report	2021	1	IgLON5	A 59-year-old woman with dysphagia, weight loss, and involuntary movements of the face and the upper limbs,visual hallucinations, episodic nocturnal confusion, and severe

	Disease						insomnia. She had diffuse myoclonus (predominant in the upper limbs, face, and tongue), dysarthria, mild parkinsonism, and cerebellar signs. Poor response to immunotherapy with frequent waxing and waning.
37.	Frequency and Characterization of Movement Disorders in Anti-IgLON5 Disease.	Gaig C et al.	Retrospective case series	2021	72	IgLON5	Dystonia was seen in 26% patients.Predominantly oromandibular or lingual dystonia (7 patients, 2 of them with painful ep-isodes of mandibular spasms that resembled trismus and prevented from normal feeding), blepharospasm (5), and cervical dystonia (3). patients had lingual and painful truncal dystonia, . Limb dystonia often occurred with cranial dystonia, with predominating involvement of the hand and fingers. Botulinum toxin had partial response.Moderate response to immunotherapy except with limb dystonia.
38.	Dystonia, lower limb stiffness, and upward gaze palsy in a patient with IgLON5 antibodies. Mov Disord. 2016	Brüggemann N et al.	Case report	2016	1	IgLON5	A 64-year-old patient , 2-year history of progressive gait impairment and lower limb stiffness, marked impairment of executive functions, apraxia, hypomimia, slow saccades with saccadic upgaze palsy. He had orolingual and mild brachial dystonia associated with an irregular dystonic tremor of the right hand while walking and mild bilateral bradykinesia.He responded well to immunotherapy.
39.	Clinical spectrum of high-titre GAD65 antibodies.	Budhram A et al.	Retrospective observational study	2021	323	GAD65	Spectrum of GAD65-associated disease is diverse including cerebellar ataxia, epilepsy, stiff person syndrome,

							myelopathy, limbic encephalitis , and cognitive dysfunction. Hyperkinetic movement disorders were considered an atypical presentation and among them dystonia and chorea were common.
40.	A unique phenotype associatedwith anti-GAD antibodies.	A.P. Mentreddi et al	MDS congress abstracts	2016	1	GAD65	A 65-year-old Indian woman, with progressive gait impairment requiring use of a walker and right-hand tremor, posturing. She had dysarthria; and right-sided rigidity, bradykinesia, rest tremor, dysmetria, dysdiadochokinesia, and dystonia. Gait was slow, ataxic and there was right foot dystonia. Moderate response to immuneotherapy.
41.	Anti–Glutamic Acid Decarboxylase Encephalitis Presenting With Choreo-Dystonic Movements and Coexisting Electrographic Seizures.	Azevedo Kauppila L et al.	Case report	2019	1	GAD 65	A 38-year-old woman presented with a 4-month history of involuntary movements of the left hand, slurred speech, and gait instability. There was moderate dysarthria and mild broad-based gait ataxia. Bilateral orofacial dyskinesia predominantly involved the left lower hemiface, lips, and fore- head. There was right laterocollis and right-shoulder elevation and proximal dystonia of the left arm and forearm. Good sustained response to IVIg and oral steroids.
42.	Stiff person spectrum disorder diagnosis, misdiagnosis, and suggested diagnostic criteria.	Chia NH, et al.	Retrospective observational	2023	173	GAD65	In a cohort of 173 cases, 48 (28%) were diagnosed with SPSD and 125 (72%) with non-SPSD. Most SPSD were seropositive (41/48: GAD65-IgG 28/41, glycine-receptor-IgG 12/41, amphiphysin-IgG 2/41).Apart from ataxia Other movement disorders (hyperekplexia, post-anoxic spasticity, dystonia) were noticed in 13%(4/29)

							cases.Misdiagnosis was threefold more common than confirmed SPSD.
43.	Intrathecal-specific glutamic acid decarboxylase antibodies at low titers in autoimmune neurological disorders.	Sunwoo JS et al.	Retrospctive observational study	2016	32	GAD 65	Two patients had dystonia-one with oromandibular dyskinesia and dystonia of the limbs with limbic encephalitis, and another blepharospasm, jaw deviation, and dystonia involving the face and neck.
44.	GAD65 neurological autoimmunity	Andrew McKeon, MD et al	Literature review	2017		GAD 65	Some patients develop cerebellar ataxia alone, while others have multifocal disorders. More unusual findings can include parkinsonism, blepharospasm, chorea, and dystonia.
45.	Glutamic acid decarboxylase autoimmunity with brainstem, extrapyramidal, and spinal cord dysfunction	Pittock SJ et al.	Retrospective observational	2006	62	GAD 65	Dystonia is rare in GAD 65, two patient had dystonia with one patient having dystonic posturing of the left arm with florid brainstem signs and another patient had blepharospasm.
46.	Anti-gamma-aminobutyric acid receptor type A encephalitis: a review.	Guo CY, Gelfand JM et al	Retrospective observational	2020	50	GABAa	Anti-GABAA receptor encephalitis can present across the age spectrum and should be considered in patients who present with rapidly progressive encephalopathy and/or seizures. Associated movement disorders, including oro-facial dyskinesias, dystonic postures, and generalized choreoathetosis, were found in 14% (7/50).
47.	Movement Disorders Associated with GABAA Receptor Encephalitis.	Vacchiano V et al	Case report	2020	1	GABAa	A 47-year-old woman with history of systemic sarcoidosis presented with sudden onset of repetitive abnormal movements in the right hand, spread after 1 day to the left hand, and then in the right lip described as dystonia.MRI showed multiple FLAIR hyperintensities. There was

							excellent response to immunotherapy (Steroids +Rituximab)
48.	GABAA receptor encephalitis associated with human parvovirus B19 virus infection	Daniel Almeida et al	Case report	2021	1	GABAa	A 6 year boy with recurrent status epilepticus , cerebellitis ,encephalopathy with MRI showing multiple FLAIR hyperintensities. He had abnormal movements characterized by right lip and right upper limb posturing without loss of consciousness even in the presence of various antiepileptic drugs. A video electroencephalogram performed during a seizure revealed a non-epileptogenic nature of the facial and upper limb myoclonic events.
49.	Investigations in GABAA receptor antibody-associated encephalitis.	Spatola M et al.	Cohort Study	2017	26	GABAa	Movement disorder were seen in 35% patients, who showed orofacial dyskinesias, dystonic postures, or generalized choreoathetosis. Usually it is common in children as young as 2.5 months and often associated with concurrent NMDA receptor positivity
50.	GABAA receptor autoimmunity: A multicenter experience	O'Connor et al	Retrospective observational	2019	4	GABAa	GABAAR encephalitis should be considered in patients with encephalopathy and seizures (especially those refractory to antiepileptic drugs) with multifocal brain lesions on imaging. Prompt immunotherapy and neoplasm search should follow
51.	Antibodies to surface dopamine-2 receptor in autoimmune movement and psychiatric disorders. Brain	Dale RC et al.	Retrospective observational	2012	22	D2R	Patients had movement disorders characterized by parkinsonism, dystonia, tics and chorea along with psychiatric disturbance with emotional lability, attention deficit and psychosis. Five of twelve (5/12) seropositive patients with dystonia had coarse dystonic tremor. Oculogyric

							crises and ocular flutter also noted. MRI showed lesions localized to the basal ganglia in 50% of the patients
52.	The neuropsychological profile of children with basal ganglia encephalitis:	Pawela C et al.	Case series	2017	4	D2R	Dystonia is a prominent movement disorder in 75% (3/4) patients which is usually isolated focal limb dystonia progressed into hemidystonia often associated with dystonic tremors. They often progress to status dystonicus.
53.	Anti-Dopamine Receptor 2 Antibody-Positive Encephalitis in Adolescent.	Dai X et al.	Case report	2020	1	D2R	A previously healthy 17-year-old Chinese girl was admitted with psychological and behavioral abnormalities, akinesia. Her MRI showed bilateral basal ganglia lesion and following IV steroids she developed retrocolis and postural tremors in the limb with raising antibody titre. She also responded well to immunotherapy.
54.	Relapsing and Immune-Responsive Paroxysmal Jaw Clonus With Blepharospasm and Sialorrhea Associated With D2R Autoantibodies.	Liu Y et al	Case report	2022	1	D2R	A 13-year-old adolescent boy was admitted with Paroxysmal jaw clonus, blepharospasm, and sialorrhea with a history of Tourette syndrome and OCD. The symptoms responded to IV methylprednisolone (IVMP), relapsed twice during prednisone reduction, and, finally, improved after the combined treatment of IVMP and IV immunoglobulin
55.	Autoimmune Encephalitis and Other Neurological Syndromes With Rare Neuronal Surface Antibodies in	Ancona C et al.	Systematic Review	2022		D2R	The most frequent neuronal surface antibody syndromes were anti-D2R (30%), anti-GABAAR (24%), and anti-GlyR (23%). The median age at onset was 5.5 years (mean 5.4, range 0.8–17). Four main clinical syndromes were identified-

	Children: A Systematic Literature Review						basal ganglia encephalitis (71.4%) (BGE), Tourette's syndrome(TS) (14.3%), isolated psychosis (10.7%), and AE (3.6%). The most frequent symptoms were movement disorders (95.2%): dystonia, chorea, parkinsonism, ocular symptoms; psychiatric symptoms (71.4%),cognitive symptoms (62.5%): MRI was abnormal in 71.4%, mostly showing basal ganglia T2 hyperintensity. No abnormalities found among TS and isolated psychosis subgroup.
56.	Autoimmune encephalitis in children: Clinical phenomenology, therapeutics, and emerging challenges.	Dale RC, Gorman MP, Lim M.	Literature review	2017		D2R	Basal ganglia encephalitis with D2 receptor antibody positivity predominantly presents with dystonia-parkinsonism and emotional lability.
57.	Post-streptococcal autoimmune dystonia with isolated bilateral striatal necrosis	Russell C Dale MRCP et al	Case series	2002	2	D2R	We describe two children children (1 year 2 months and 4 years) with the bilateral striatal necrosis who presented shortly after streptococcal pharyngitis. Both patients had autoantibodies reactive against components of the basal ganglia.Other anti-neuronal antibodies were negative, supporting striatal specificity .As control, 20 children with dystonia , and 20 children with uncomplicated streptococcal infection were examined.
58.	Anti-dopamine D2 receptor antibodies in chronic tic disorders:	Addabbo F et al the EMTICS Collaborative Group	Prospective longituidinal observational study	2019	715	D2R	At baseline, 9/137 (6.6%) study participants exhibited anti-D2R positive sera. At the time point of tic exacerbation, 20/137 subjects (14.6%) had anti-

	potential link to fluctuations of tic severity						D2R positive sera, with newly seroconverted, 8%. Presence of antiD2R antibodies in serum was significantly associated with exacerbation visits when compared to baseline visit.
59.	Neuroimmunological antibody-mediated encephalitis and implications for diagnosis and therapy in neuropsychiatry.	Marinas JE et al	Literature review	2019		D2R	D2 receptor encephalitis usually occurs in childhood, affecting both sexes equally (Dale et al., 2012). This condition is mostly seen after infection with β-hemolytic streptococcus, mycoplasma and entero-virus as well as after vaccination. MRI lesions are seen in the basal ganglia in approximately 50% of patients. Clinical presentation involves dystonia and oculogyric crises, features of parkinsonism and chorea.
60.	Phosphodiesterase 10A IgG A novel biomarker of paraneoplastic neurologic autoimmunity	Anastasia Zekeridou, MD et al	Case series	2019	7	PDE 10A	The median patient age was 70 years (range 66–76); 4 were men. Four patients had movement disorders (hyperkinetic in 3 [chorea, ballismus, dystonia] and parkinsonism in 1). All patients but one had cancer.
61.	Atypical neurological syndromes in anti-MA2 associated encephalitis: Two case reports	Sruthi Degapudi et al	Case reports	2023	2	Anti Ma2	A 26 year old male presented with a 1-month history of diplopia, cognitive dysfunction and somnolence with difficulty in jaw opening. Examination revealed upgaze palsy, convergence retraction nystagmus, jaw-closing dystonia, and parkinsonism. MRI revealed T2 FLAIR hyperintensities in bilateral medial thalamus, hypothalamus, bilateral medial temporal lobe, dorsal midbrain and dorsal pons consistent with limbic/brainstem encephalitis. Patient had an enlarged left testis but he

							succumbed to severe pneumonia prior to completion of malignancy screening
62.	A case of an anti-Ma2 antibody-positive patient presenting with variable CNS symptoms mimicking multiple system atrophy with a partial response to immunotherapy] [Article in Japanese]	Wataru Shiraishi [1], Yasutaka Iwanaga, A kifumi Yamamoto	Case Report	2015	1	Anti Ma2	A70-year-old male presented with rapidly progressive symmetric akinetic rigid parkinsonism, left upper limb dystonic posturing, and orthostatic hypotension. His cranial and spinal MRI and cerebrospinal fluid test were unremarkable, however, he had Anti Ma2 antibody positivity in serum. He was treated with high-dose methyl prednisolone resulting in improvement in his symptoms
63.	Paraneoplastic Chorea Associated with CRMP-5 Neuronal Antibody and Lung Carcinoma,	Steven Vernino et al	Case Series	2002	16	Anti CRMP5	They described 16 patients who presented with chorea, and 4 of them had dystonic posturing. All of them had combined dystonia mostly limited to the affected limbs
64.	Anti-Myelin Oligodendrocyte Glycoprotein (MOG) antibody disease presenting with severe dystonia	Tuba Rashid Khan et al	Case Report	2021	1	Anti MOG	A 3-year-old boy presented with new onset seizure preceded by malaise and fever. MRI showed multifocal brain and spinal cord lesions.He developed severe orofacial and limb dystonia. CSF findings were consistent with MOGAD as there was mild lymphocytic pleocytosis with normal to mildly elevated protein and no oligoclonal bands.Responded well to immunotherapy, baclofen, Botox injection.
65.	Paediatric	Mario Sa et	Case	2018	1	Anti	We describe a 2-year-9-

	MOG antibody–associated ADEM with complex movement disorder: A case report	al	Report			MOG	month-old girl who presented with severe encephalopathy with aphasia, seizures and a complex movement disorder with dystonic posturing and tonic eye deviation. Neuroimaging revealed subtle asymmetrical predominantly white matter signal changes. MOG-Abs were positive in the serum. The patient made a complete recovery following 2-week corticosteroid treatment.
66.	Myelin oligodendrocyte glycoprotein antibody-associated disease presenting with dystonia	Omri Zveik Et al	Case Report	2023	1	Anti-MOG	A 28-year-old female presented with right-arm dystonic episodes. MRI demonstrated multifocal periventricular and subcortical lesions, including in the left centrum semiovale and corona radiata. Anti MOG was positive and she responded well with immunotherapy.
67.	Encephalitis with mGluR5 antibodies Symptoms and antibody effects	Marianna Spatola, MD et al	Case Series	2018	11	Anti mGluR5	Anti-mGluR5 encephalitis associates with a complex neuropsychiatric syndrome, not restricted to limbic encephalitis. Two patients had dystonia associated with generalised seizure, cognitive, sleep disturbances.
68.	Anti-metabolic glutamate receptor 5 encephalitis with gangliocytoma: a case and review of the literature	Kaili Shi et al	Case Report	2024	1	Anti mGluR5	A 16 year female with Hodgkin lymphoma and psychosis, hallucinations, poor sleep, dystonia, generalized seizures treated with steroids and Rituximab and had partial response

69.	Autoimmune encephalitis with mGluR5 antibodies: A case series from China and review of the literature	Kundian Guo et al	Case Series	2023	15	Anti mGluR5	A 35 year female with acute onset spatial disorientation, prosopagnosia, memory deficits visual hallucination, generalized seizures, refractory status epilepticus, then rapidly progressive decreased level of consciousness and being in a coma, dystonia, hypoventilation.
70.	Movement disorders in systemic autoimmune diseases: Clinical spectrum, ancillary investigations, pathophysiological considerations	Menozzi E etal	Systematic Review	2021		Sjogren Syndrome	Two phenotypes of primary Sjogren Syndrome(PSS)- 1.dystonia - In 50% of cases;characterised by subacute and slowly progressive focal or segmental dystonia predominantly affecting the craniocervical region. Brain MRI is often normal . The response to immunotherapy is satisfactory.2. The second phenotype, occurring in younger individuals (median age at onset 47 years; 75% female) consisted of paroxysmal(< 2mintes, painful with high frequency) dystonic attacks involving one or both limbs on one side of the body with inflammatory and/or ischaemic lesions were detected either in the brain or spinal cord . CSF was normal in ¾ of these cases .
71.	Neurologic involvement in seronegative primary Sjögren's syndrome with positive minor salivary gland biopsy: a single-center experience.	Hoshina Y et al.	Retrospective observational	2023	45	Seronegative Sjogren Syndrom	Neurologic manifestations of seronegative PSS are heterogeneous with neuropathy being the most common presentation and 2.2% presenting with dystonia.
72.	Bilateral	Kargiotis O	Case	2010	1	Seroneg	58-year-old Caucasian

	blepharospasm as the presenting symptom of Sjögren's syndrome with evidence of central nervous system involvement.	et al	report			ative Sjogren Syndrome(Biopsy positive)	woman who was admitted with progressive blepharospasm previous 3 months. MRI brain showed high signal in T2/FLAIR in basal ganglia , thalamus,minor salivary gland biopsy consistent with PSS. She also had mild xerostamia. Responded moderately with methyl prednisolone after a poor response to Inj Botox.
73.	Efficacy of high dose methylprednisolone in a patient with cervical dystonia and blepharospasm and Sjögren's syndrome.	Mantero V et al	Letter to editor	2015	1	Seronegative Sjogren syndrome(Biopsy Positive)	A 67-year-old woman with cervical dystonia with anterior flexion of the neck, and blepharospasm for 3 months with a previous H/O Sjogren syndrome 5 years back. MRI Brain was normal,Had rapid and sustained response to IV steroid
74.	Orofacial dystonia related to Sjogren's syndrome. Clin Rheumatol	Papageorgiou SG et al	Case report	2007	1	Seronegative Sjogren Syndrome(Biopsy positive)	A 57-year-old woman presented with orofacial dystonia that had appeared 2 months previously. Since 2 years, she had spasmodic dysphonia ,blepharospasm with involuntary movement extending to the whole face and neck. She is K/C/O Sjogren syndrome with marked xerostomia, raised antinuclear factor antibodies (1:2,560), and a positive salivary gland biopsy. Anti-Ro and anti-La antibodies were negative.MRI was normal.She had marked improvement with IV steroids followed by oral steroids.
75.	Case of primary Sjogren's syndrome preceded by dystonia	Kerime Ararat et al	Case report	2018	1	Sjogren syndrome	43-year-old woman who presented initially with left hemidystonia , sensory neurogenic bladder, migraine like headaches. She also had Raynaud's phenomenon, fatigue and depression. The dystonic

							movements showed as curling of her hands with hand grips and flexion of her left elbow along with curling on her left foot, and sometimes the right foot. Movements were spontaneous and stimulus induced.MRI Brain showed subtle frontal FLAIR signal changes.ANA positive, RF positive, Anti SSA and SSB positive. Responded to IV steroids followed by cyclophosphamide infusion followed by azathioprime and Inj Botox.
76.	Painful Tonic / Dystonic Spasms in Sjogren ' s Syndrome.	Jabbari B, Salardini A.	Case Series	1999	3	Sjogren Syndrome	Case 1- 53 yr male with left optic neuritis, left hemiparesis with hemianesthesia upto T2 dermatome, left foot drop, with normal Brain and Spine MRI. Anti SSA and SSB positive. Developed acute painful, tonic spasm responded to steroids.<br>Case 2- 41 year female with dry eyes, right upper limb tonic spasm. MRI cervical spine showed spinal cord infarction and venogram showed transverse and sigmoid sinus thrombosis. Anti SSA positive ,ANA speckled. Had good improvement with steroids.<br>Case 3- 36 year female with transverse myelitis and diagnosed Sjogren syndrome presented with painful tonic spasm in both lower limb.MRI showed signal changes in T2 – T7. Poor response to CBZ and baclofen.
77.	Facial reflex hyperexcitability in geniospasm suggests a brainstem origin	Aggarwal A, et al	Case Report	2009	1	Sjogren Syndrome	The bilateral mentalis activity and hyperex-citability of mentalis subnuclei suggests that geniospasm is likely to originate from loss of inhibition or hyperexcitability of central projections to the facial

							nuclei
78.	Sjogren's syndrome meets Meige's syndrome	Xuemei Li et al	Case report	2023	1	Sjogren Syndrom	A 73-year-old male with dry eye symptoms for the past 5 years and eyelid spasms and tetanic eye closure occurring 3 years ago. Traditional treatments, including subthalamic nucleus deep brain stimulation, provided only temporary relief. Diagnostic evaluations, confirmed Sjogren syndrome and Multiple Sclerosis coexistence. Treatment involved a combination of steroids, immunosuppressants, and immunoglobulin, leading to significant symptom relief.
79.	Neuroimaging Pearls from the MDS Congress Video Challenge. Part 2	Fearon C, et al.	MDS congress presentation	2022	1	Sjogren Syndrome	A 23 year-old woman presented with a one-month history of intermittent, involuntary, painful tonic flexion of both wrists and MCP joints. Two months prior, she had holocranial headaches, hiccups, and intractable vomiting. Examination revealed left hemi-sensory loss, bilateral brisk deep tendon reflexes, and upgoing plantars. CSF aquaporin-4 antibodies were negative. Anti Ro was strongly positive. MRI spine confluent T2 hyperintensity from C2 to C4/C5 without expansion of the cord.
80.	Juvenile parkinsonism as a manifestation of systemic lupus erythematosus : Case report and review of the literature.	García-Moreno JM et al	Case Report	2002	1	SLE	A 20-year-old, left-handed, female patient with bradykinesia and painful dystonia of the left arm , resting tremor in the left leg, and dystonic postures of both feet. ANA positive with a speckled pattern at the titer of 1:320, Anti-ds DNA antibody positive (23.8 U/ml; normal < 6 U/ml), C4 hypocomplementemia , IgG and IgM anticardiolipin

							antibodies were positive at low titers. Good sustained response after steroids , levodopa,bromocriptine and VIM DBS.
81.	Bilateral reversible basal ganglia changes associated with dystonia and hemifacial spasms in central nervous system lupus.	Wu K et al	Case Report	2015	1	SLE	A 40-year-old woman with (SLE) and associated inflammatory polyarthritis who presented with acute facial dystonic spasms.. An MRI brain showed bilateral symmetrical basal ganglia signal change on T2. Her symptoms resolved rapidly following treatment with oral steroids. Repeat MRI brain at 1 month showed complete resolution of the basal ganglia signal changes.
82.	Movement disorders in systemic lupus erythematosus and antiphospholipid syndrome - A video presentation	Ramcharan K et al	Case Report	2015	1	SLE	An 18 year boy with acute onset myoclonus in right upper limb, facial muscles,choreoathetoid movements of the limbs and feet dystonia. C3 and C4 were low. ANA and Anti dsDNA were strongly positive, and histones and ribosomes++, β2-GP1 IgG and IgM, anticardiolipin IgG, A and M and lupus anticoagulant were markedly elevated. MRI multiple cortical and subcortical lesion. He responded well to IV steroids followed by oral steroids and mycophenolate.
83.	Jaw Dystonia and Reversible Basal Ganglia Changes as an Initial Presentation of Systemic Lupus Erythematosus	Romba M, et al.	Case report	2018	1	SLE	We report a 25-year-old woman who experienced progressive confusion, reduced speech, and jaw dystonia 2 weeks after development of a facial rash. Brain imaging showed bilateral, symmetric signal abnormalities within the basal ganglia and sub-cortical white matter. Both clinical and radiological resolution with IV steroids and the initiation of

							levodopa.
84.	Bilateral reversible basal ganglia changes associated with dystonia and hemifacial spasms in central nervous system lupus	Kit Wu	Case Report	2015	1	SLE	We report a 40-year-old woman with systemic lupus erythematosus (SLE) and associated inflammatory polyarthritis who presented with acute facial dystonic spasms. Her speech was also affected. An MRI brain showed bilateral symmetrical basal ganglia signal change on T2. Her symptoms resolved rapidly following treatment with (oral) steroids. Repeat MRI brain at 1 month showed complete resolution of the basal ganglia signal change.
85.	Systemic lupus erythematosus presenting with steroid-responsive parkinsonism and post-hemiplegic dystonia	SM Lim et al	Case report	1998	1	SLE	A young male patient who presented with steroid-responsive parkinsonism, hemiplegia, thrombocytopaenia and systemic illness. He later developed -hemidystonia with thalamic lesions on magnetic resonance imaging (MRI).
86.	Writer's Cramp and Psychosis: An Atypical Presentation of Systemic Lupus Erythematosus	Roy* JNG and S	Case Report	2021	1	SLE	A 9-year-old right-handed girl presented with handwriting deterioration and behavioral issues for five months. Initially, she had painful grip tightening and writing difficulty after writing about ten lines, which resolved on resting. After a month, symptoms emerged immediately on writing few words. Serum ANA: strongly positive (speckled); Anti dsDNA:positive; C3 and C4 levels: low;APLA : absent. Had good response to IV steroids and oral steroids , THP.
87.	Adult onset dystonia of the foot as a presenting symptom of	Elena Glebovna et al	Case Report	2011	1	SLE	A 31-year old female patient with a 3 year history of a focal right foot and left arm dystonia . Treatment w

	systemic lupus erythematosus						ith tetrabenazine and botuli num toxin was moderately effective.<br>Brain and spinal cord MR, dopamine transporter SPEC T were normal. Laboratory workup included a positive ANA 1/80 (speckled pattern), positive ds-<br>DNA, positive anti-<br>nucleosome antibody, positi ve anti-TPO, anti-<br>TG antibodies, with normal complement,<br>and a normal PCR for DYT -1 deletion gen TOR1-<br>A. Two months later the pat ient developed<br>poliarthritis, asthenia, malar rash, oral ulcers, psychosis and depression for which a diagnosis of SLE was made and a trial of steroids, chlor oquine, antidepressant and antipsychotic started with m oderate improvement.
88.	Torticollis and blepharospas m in systemic lupus erythematosus	Rajagopala n N, et al	Case Report	1989	1	SLE	A 56-year-old woman presented with blepharospasrn followed by torticollis. She presented with fever and joint pains later with ANA titer 800 IU/ml(1:10) and of homogenous and nucleolar staining type. The latex test for rheumatoid factor was positive. Complement levels (C3,C4) were normal. There was no evidence of lupus anticoagulant, the kaolin cephalin clotting time being normal.MRI was normal. She responded well to steroids during exacerbation of blepharospasm with raising antibody titre.
89.	Blepharospas m and Autoimmune Diseases1	Joseph Jankovic and Bernard M. Patten	Case Report	1987	1	SLE	A 40-year-old right-handed woman with a 7-year history of skin rashes, dermatitis, pruritic nodules, arthralgias, myalgias, fatigability, diagnosed to have SLE with positive skin

							biopsy and high titers of ANA and anti-dsDNA antibody. She developed blepharospasm and generalised myalgia which responded after IV steroids and cyclophosphamide.
90.	Neuropsychiatric Manifestations of Antiphospholipid Syndrome	Man YL, Sanna G.	Narrative review	2022	1	APS	Patients commonly present with focal neurological issues like transient ischaemic attacks and ischaemic strokes, with identifiable lesions on brain imaging or diffuse brain dysfunction like cognitive dysfunction, seizures, headache and movement disorders like chorea.
91.	Antiphospholipid syndrome and dystonia-parkinsonism. A case report.	Milanov I, Bogdanova D.	Case Report	2001	1	APS	A 60 year old, right-handed man with writer's cramp, bradykinesia and stiffness of his right hand. Neurological examination revealed dystonic posturing, rigidity and bradykinesia of the right hand. Anticardiolipin antibodies IgM was positive. There was also slight thrombocytopenia. MRI revealed several hyperintense lesions in the basal ganglia and in the periventricular white matter and subcortical white matter with asymmetric parenchymal atrophy. No clinical improvement was achieved by levodopa, dopamine agonists or anticholinergics.
92.	Antiphospholipid syndrome presenting with craniocervical dystonia: A case study.	C. Dietiker et al	Case Report	2017	1	APS	A 76 year female with dementia, hallucination, and gait abnormality exhibited orofacial dystonic movements including jaw opening, lip pursing, and blepharospasm. She had left torticollis and phasic shoulder elevation bilaterally, as well as continual agitated and semi-purposeful movements in her limbs that did not

							appear choreiform. MRI brain at admission showed subacute strokes in the left caudate and bilateral centrum semiovale. She was diagnosed with APS after laboratory studies revealed positive cardiolipin and beta-2-glycoprotein antibodies as well as positive lupus anticoagulant. She was treated with IV solumedrol and transitioned to prednisone and mycophenolate mofetil, with marked improvement of her dystonia.
93.	Hemidystonia symptomatic of primary antiphospholipid syndrome in childhood.	Angelini L et al	Case Series	1993	3	APS	Case 1- A 7 year female with left sided hemidystonia, hemiballism with MRI showing diffuse signal hyperintensity in the periventricular white matter of the right hemisphere and the posterior portion of the internal capsule. A high titer of anti- cardiolipin (aCL) antibodies (27 U IgG) was detected. She responded well to oral steroids.<br>Case 2- A 12 year boy with rapid onset encephalopathy, left facial chorea,left hemidystonia.MRI showed right putamen infarct. aCL antibodies present at high titer (23 U IgG).Clinical and radiological resolution without steroids.<br>Case 3- A 14 year girl with perinatal injury with right hemidystonia from age 2,MRI at 12 years showed left putaminal and caudate infarct. LA was normal, but aCL antibodies (9.3 U IgG) were demonstrated.
94.	Antiphospholipid antibodies and cerebellar ataxia: A clinical analysis and	Chen WH et al	Case Series	2014	4	APS	One patient (1/4) had dystonia. Case 4 – 51 year female with residual left hemiparesis with ataxia, generalized chorea and dystonia. β2GPI was

	literature review.						positive.All movement disorders improved with PLEX.
95.	Movement Disorders in Antiphospholipid Syndrome & Systemic Lupus Erythematosus	Danielle S. et al	Literature Review	2020		APS and SLE	A variety of movement disorders have been recognized in the context of APS and SLE, although chorea is by far the most commonly reported. Although many cases have been associated with other movement disorders like ataxia , dystonia, parkinsonism, myoclonus.Ischemic-appearing lesions of the basal ganglia and surrounding structures, many have occurred in the context of normal or nonspecific brain imaging, suggesting a more complicated pathophysiologic mechanism.
96.	Complex movement disorders in primary antiphospholipid syndrome: A case report	Miryam Carecchio	Case Report	2008	1	APS	79 year female with insomnia, non fluent aphasia, encephalopathy, chorea, orolingual dyskinesia,left foot focal dystonia.MRI showed left post temporal lobe infarct. The diluted Russel Viper Venom test (DRVTT) for lupus anticoagulant (LAC) was positive and a strongly increased anticardiolipin (aCL) IgG titer (N91 GPL/ml; normal b14) was found. IgG and IgM anti-β2 glycoprotein-I titer was considerably high, too (95.5 U/ml; normal b14; 45,2 U/l; normal b17).She significantly improved with warfarin and steroids.
97.	Neurologic Manifestations of the Antiphospholipid Syndrome — an Update	Leal Rato M, Bandeira M, Romão VC, Aguiar de Sousa D.	Literature review	2021		APS	Although stroke is the most common cause of neurological manifestations in patients with APS, other neurological disorders have been increasingly associated with the disease, including cognitive dysfunction, headache, and

							epilepsy.Among movement disorders chorea is the commonest and it is often associated with other neurologic and movement disorders such as ataxia and dystonia.
98.	Antiphospholipid Antibody-Associated Chorea	Nicole m. Orzechowski et al	Retrospective observational study	2008	18	APS	aPL-associated chorea occurs most often in women and severity is mild to moderate. Clinical expression of chorea does not differ between those with and without SLE. 2 out of 18 patients had mild dystonia.
99.	Neuro-Behçet's disease: epidemiology, clinical characteristics, and management.	Al-Araji A, Kidd DP.	Systematic Review	2009	1013	NBD	In neuro-BD (NBD), the CNS can be involved in one or both of two ways: first, and most commonly, through the development of an immune- mediated meningoencephalitis, which predominantly involves the brainstem, but can also involve the basal ganglia, thalamus, cortex and white matter, spinal cord, or cranial nerves; and second, because of thrombosis within the dural venous sinuses.
100.	Clinical patterns of neurological involvement in Behcet's disease: Evaluation of 200 patients.	Akman-Demir G, Serdaroglu P, Tasçi B.	Retrospective observation	1999	200	NBD	Neuro-Behcet's disease is a common multisystemic disorder with a predilection for the brainstem and basal ganglion region. Among 200 cases (155 male, 45 female) evaluated: 162 had parenchymal CNS involvement (brainstem or 'brainstem +' involvement in 51%, spinal cord involvement in 14%, hemispheric involvement in 15% and isolated pyramidal signs in 19%). Movement disorders such as hemichorea, hemiballismus and hemidystonia were seen in 6%.
101.	Characteristics of	Houman MH et al.	Retrospective	2013	430	NBD	Neurological involvement was observed in 121

	neurological manifestations of Behçet’s disease: A retrospective monocentric study in Tunisia		observational				patients (28.1%). Among them 26 (21.4%) presented with brainstem involvement, 24 (19.8%) with hemispheric involvement and 2 (1.6%) with spinal cord involvement. Male gender and CNS parenchymal lesions occurrence were significantly associated with a poorer prognosis
102.	Paroxysmal Focal Dystonia in Neuro-Behcet by a Small Ipsilateral Thalamic-Lesion	Tae-Ho et al	Case Report	2002	1	NBD	A 21-year-old woman was diagnosed 3 years ago as Behcet disease. She complained of a sudden irregular throwing-like movement of the right arm which was followed by dystonic state in the right arm and in the right perioral areas within 24 h. These attacks were sudden, lasting 15-30 s and occurring 10-20 times a day.MRI right thalamic T2 hyperintensity. Other work ups negative. She was prescribed 10 mg prednisolone and had good response.
103.	Hemibody dystonia secondary to Neurobehçet disease : a case report	S. Saaf, Z. El et al	Case Report	2023	1	NBD	A 29 year patient, with a history of neuro-behçet disease treated with monthly IV cyclophosphamide, presented with painful, cramp-like, contortions evoking right hemibody dystonia with a right hemiparesis rated at 3/5 was found, as well as a spastic hyperreflexia with crossed reflexes and oro-facial apraxia and aphasia. Brain MRI showed a hyperintense signal in T2 FLAIR sequence on the left putamen and the caudate nucleus. EEG showed IEDs. Treated with carbamazepine and baclofen with good response.

Abbreviations

aCL = Anti cardiolipin antibody

ANA = Anti-Nuclear Antibody

Anti ANNA 2 = Antineuronal Nuclear Autoantibody Type 2 (Anti-Ri).

Anti ds- DNA = Anti double stranded DNA

Anti TG = Anti Thyroglobulin

Anti TPO = Anti Thyroid Peroxidase

Anti β2-GP1= Anti β2 Glycoprotein 1

APAbs = Anti Phospholipid antibody

APS= Anti Phospholipid Syndrome

CSF= Cerebrospinal Fluid

CTD= Connective Tissue diseases

D2R= Dopamine-2 receptor

EEG = Electroencephalogram

FBDS= Faciobrachial Dystonic Seizures

FLAIR=Fluid-attenuated inversion recovery

GAD65 Encephalitis = Anti-glutamic acid decarboxylase 65 associated encephalitis

IgLon5 Encephalitis =Anti Immunoglobulin-like cell adhesion molecule 5 associated encephalitis

IV= Intravenous

LA = Lupus Anticoagulant

LGI1 Encephalitis = Leucine-rich glioma-inactivated 1 associated encephalitis

MEG = Magnetoelectrogram

MRI = Magnetic Resonance Imaging

NBD = Neuro Behcet's Disease

NMDA encephalitis= Anti-*N*-methyl-d-aspartate receptor encephalitis

OFLD= orofacial lingual dyskinesia

PET = Positron Emission Tomography

PSS = Primary Sjogren Syndrome

SPECT=Single-photon emission computed tomography

SPSD = Stiff person spectrum disorder
